# Upcycling of Beer Processing Residues: Insights for Microbreweries Sustainability

**DOI:** 10.1021/acsomega.5c09979

**Published:** 2026-01-23

**Authors:** Gustavo Henrique Couto, Débora Gonçalves Bortolini, Emanuele Elisa Hernandes, Elisabete Hiromi Hashimoto, Deborah Lizama Boettcher, Sabrina Ávila Rodrigues, Mário Antônio Alves da Cunha, Maria Giovana Binder Pagnoncelli

**Affiliations:** † 74354 Universidade Tecnológica Federal do Paraná (UTFPR), Postgraduate Program in Biotechnology (PPGBIOTEC), Ponta Grossa, Paraná, CEP 84016-210, Brazil; ‡ Universidade Tecnológica Federal do Paraná (UTFPR), Department of Chemistry, Via do Conhecimento km 01, Pato Branco, Paraná 85503-390, Brazil; § Universidade Tecnológica Federal do Paraná (UTFPR), Postgraduate Program in Biotechnology (PPGBIOTEC), Department of Chemistry and Biology, Rua Deputado Heitor Alencar Furtado 500, Curitiba, Paraná 81280-340, Brazil; ∥ Centro Universitário FAEL (UniFAEL), Rodovia Olívio Belich, km 30, Lapa, Paraná 83750-000, Brazil

## Abstract

The rapid growth of the microbrewery sector, driven by the increasing demand for craft beers, has highlighted the importance of managing brewing residues to ensure environmental and economic sustainability. This review explores the primary byproducts of beer production—brewer’s spent grain (BSG), brewer’s spent yeast (BSY), and hot trub—analyzing their composition and potential for upcycling. By exploring the Brazilian microbrewery sector as a case study, this work highlights its growth and challenges, while drawing insights that resonate with the global interest in sustainable practices within the craft beer industry. Traditional applications, such as animal feed and fertilizer, are expanded with new approaches like biofuel production, biogas, biopolymer synthesis, prebiotics, and healthy food ingredient development. An emphasis on the applications of BSG in food formulations, such as bread, cookies, nutrition bars, beverages, meat analogs, yogurt, and others, highlights its nutritional value and functional properties for developing food products. By integrating sustainable practices and advanced processing technologies, brewing residues can be transformed into high-value products, reducing waste and fostering economic growth. This review concludes that adopting circular economy principles is essential for aligning microbrewery operations with global sustainability goals while unlocking the potential of brewing byproducts for diverse industrial applications.

## Graphical abstract

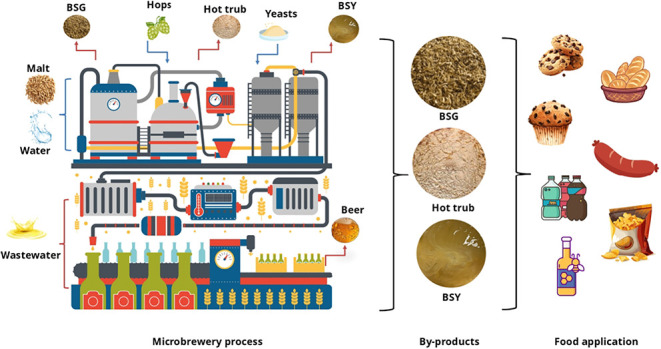

## 1 Introduction

The microbrewery sector has experienced remarkable global growth, highlighted by increasing consumer demand for craft products and unique flavors. In several countries, this industry is flourishing, significantly contributing to local economies while fostering the diversification of beer styles.^1–3^ This review addresses strategies for managing the substantial volume of waste generated during beer production.^4,5^ Beer production involves a series of unitary operations that generate solid residues, including brewer’s spent grain (BSG) or malt bagasse, brewer’s spent yeast (BSY), hot trub; and liquid waste such as wastewater from equipment cleaning, process water from cooling and pasteurization, and fermentation effluents.^6,7^

These solid byproducts are produced in notably large amounts. Each 100 L of beer is estimated to 25 ± 5 kg of BSG, 3 ± 1 kg of BSY, and 0.3 ± 0.1 kg of hot trub.^8,9^ If not managed properly, these byproducts pose environmental risks. At the same time, they offer potential for reuse in various applications, particularly in food production, due to their high nutrient content.^10,11^ Traditionally, brewery byproducts have been used in animal feed and fertilizers, with low revenue for beer producers. Indeed, their high moisture and nutrient content make them prone to microbial degradation, complicating storage and limiting broader applications.^12^

Over recent decades, the global beer industry has been reshaped by the so-called craft beer renaissance, a movement that originated in the United States during the 1970s.^13^ However, prior reviews emphasize large-scale brewing byproducts and their models for BSG valorization which are economically unfeasible for microbreweries. They do not adequately address the technical, economic, and environmental specificities of microbrewery operations, specifically constraints: limited production volumes, resource scarcity, and proximity to local communities. This review addresses that gap by providing a focused assessment of waste characterization and sustainable applications at the microbrewery scale, examining the environmental impact of brewery waste and exploring innovative uses for these byproducts. As a case study, this theme is explored more deeply through the context of Brazilian microbreweries, a sector that has experienced high local growth in recent decades. An analysis of the environmental impacts of brewing waste and an investigation into advanced applications of these byproducts, focusing on food technology and circular economy principles, are given.

## 2 An Overview of the Microbrewery Sector in Brazil

Beer, one of the oldest alcoholic beverages, is the third most widely consumed beverage worldwide after water and tea. In 2023, the global beer market was valued at approximately USD 1.1 trillion, and it is projected to grow at a compound annual growth rate (CAGR) of about 6.5% between 2024 and 2030.^14^ Small independent breweries generally produce craft or artisanal beer. It was valued at USD 102.15 billion in 2023 and is expected to grow from USD 108.8 billion in 2024 to USD 275.76 billion by 2032, with a compound annual growth rate (CAGR) of 12.33% over the forecast period (2024–2032). The U.S.-based Brewers Association reported that craft breweries hit a record high of 9,552 in 2023.^15^ The rising success of small brewers (microbreweries and brewpubs) reflects a growing appreciation for the opportunities hops provide, particularly in crafting diverse hop aromas and flavors.^3,16^ A small brewery that sells off-site at least 75% of its fewer than 15,000 manufactured barrels of beer annually is considered a microbrewery. These breweries typically craft unique and high-quality beers through traditional brewing techniques, distributing their products through various channels, including the three-tier system (brewer to wholesaler, wholesaler to retailer, retailer to consumer), a two-tier system (brewer to retailer, then to consumer), or directly to consumers through carry-out sales, taprooms, or on-site restaurants.^2^

Traditionally dominated by large-scale beer producers before the nineties, Brazil’s beer market shifted toward craft beer in the 90s when the first microbrewers emerged. In general, their founders sought inspiration in the United States and other European countries, where craft beer was rising along with increasing consumer demand for craft beers with unique flavors and high-quality standards.^5^ In the 2000s, craft breweries grew exponentially in Brazil, stimulated by events like festivals and beer competitions that moved the market.^17^

Since 2010, Brazil has experienced an explosion in the sector, producing and consuming diverse types of crafted beer, becoming a key player in the country’s evolving beer culture. According to the Brazilian Craft Beer Association,^1^ the number of microbreweries in the country has grown from just over a hundred at the beginning of the 2000s to more than 1500 establishments in 2023.

On average, Brazil has one registered brewery for every 109,952 inhabitants, and the state with the highest number of registered breweries is São Paulo state, which has 410 establishments.^18^ However, considering the number of breweries per capita, the Rio Grande do Sul fills the top position, with one establishment for every 32,486 residents. Followed by 33,8000 inhabitants per brewery in Santa Catarina, 46,700 in Espírito Santo, 66,900 in Paraná, and 87,400 in Minas Gerais.^19^ Craft breweries, which include microbreweries, account for approximately 1% of the total beer volume and around 2.5% of sales revenue in Brazil.^20,21^ Most of these breweries are micro to small-sized enterprises with limited production capacities, yet they stand out for their innovation and close connection with consumers.

### 2.1 Challenges of the Microbrewery Sector

Despite their growth, Brazilian microbreweries encounter several challenges that can be reflected in the global sector. Key issues include regulatory hurdles, differentiated zone fees, production and distribution costs, and difficulties in market positioning. One significant obstacle is the complexity of the regulatory landscape. Establishing and operating a microbrewery requires adherence to various legal obligations, which can differ by region and operational type, and acquiring licenses and permits can be time-consuming and expensive, particularly for small business owners.

Another challenge is competing against major beer brands. Large companies and giants have invested heavily in expanding so-called unique labels, attacking with new flavors and unusual ingredients. Possessing significantly more financial, logistical, and marketing resources enables them to dominate the market, maintaining a strong presence at retail locations. Acquisition processes have become common, where many successful small breweries have been acquired by multinational groups looking to expand their portfolios and tap into the growing demand for premium and specialty beers. Regarding fees, the Brazilian government has applied incentives for small producers with partial or total exemptions on specific taxes and the option to enroll in a simplified tax regime that helps boost the sector.^22^ Therefore, microbreweries constantly find ways to differentiate themselves and authentically engage with consumers.^21^

## 3 From Beer Production to Waste Management

### 3.1 Beer Production: General Aspects

Beer is an alcoholic beverage produced through yeast fermentation with *Saccharomyces* spp. (e.g., *Saccharomyces cerevisiae* for ales, *Saccharomyces pastorianus* for lagers) of a starch-based material. The manufacturing process involves several stages: milling, mashing, filtration, boiling, cooling, fermentation, maturation, and packaging. The stages of the production process are illustrated in Figure 1. The ingredients may vary depending on the type of beer being produced; malt, hops, and water are generally used.^23^ Hop additions vary strategically by brewing stage, with critical implications for trub composition. Bittering hops, typically added during the wort boil (60–90 min), undergo α-acid isomerization to produce bitterness, minimizing particulate carryover. Dry-hop additions postfermentation contribute plant matter and suspended solids, thus determining the appropriate clarification and solid-separation operation methods (e.g., cold settling, diatomaceous earth filtration, or centrifugation). Water is essential in beer production, being applied in all process stages and consisting of more than 90% of the final product. Therefore, it must have specific characteristics, such as potability, odorlessness, and flavorlessness, and be free from suspended particles and microorganisms.^24^ Additionally, the source water should contain appropriate mineral levels, such as calcium is essential for the stability and flavor of the beer.^25^ However, it is important to clarify that the mash/wort pH (approximately 5.2–5.6) is the key control point in brewing; the source water pH (typically 6.5–7.0) is less critical as long as alkalinity and mineral composition are properly adjusted.

**Figure 1:**
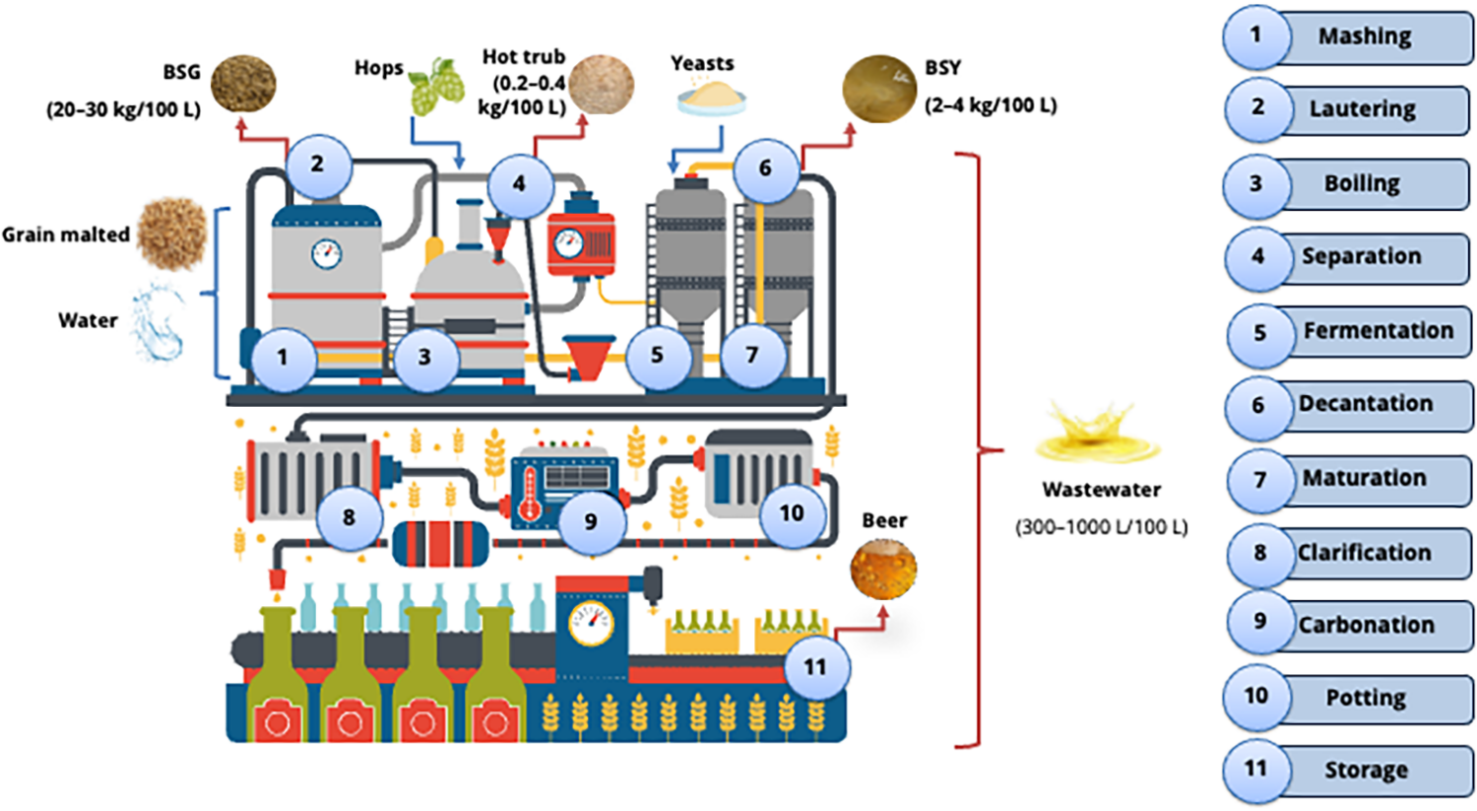
Beer production process and byproducts generation.

Beyond barley, the malt used in beer production can be derived from various other raw materials, such as rice, buckwheat, or oats. These alternative grains contribute distinct flavors, textures, and characteristics to the final product and can be used for gluten-free beer manufacturing.^26^ Malt is ground in a roller mill, which exposes the starchy content and reduces the size of the starch granules, facilitating hydrolysis in the subsequent stage.^25^ In the mashing tank, the malt is mixed with water and undergoes a cooking process, where fermentable sugars are extracted, resulting in wort. This wort then goes through the filtration stage to remove the malt husk, which consists of insoluble solids such as grain residues and malt husks.^25^ Lautering separates the wort from the spent grain, extracting fermentable sugars with hot water sprayed over the grain (sparging), resulting in a clarified wort for the next brewing stage. The residue obtained in filtration consists of BSG (20–30 kg of BSG/100 L of beer), which is rich in dietary fiber, protein, fatty acids, and minerals.^27^ Also, it is considered a phenolic-rich byproduct, which confers important biological properties.^28–30^

The filtered wort is then boiled, and hops are added to give the beer a characteristic bitter aroma and preserve the beverage due to the phenolic compounds with antioxidant properties in their composition.^31^ The amount of hops added during beer production varies according to the style, ranging from approximately 1 g/L in Pilsner to more than 10 g/L in IPA. In the microbrewery context, however, these values can be even higher, reflecting the distinctive beer styles typically produced on microbrewing. During boiling, hops may be added at different stages. Early boil hop additions promote the isomerization of α acids, increasing wort bitterness and contributing isomerized resins that precipitate together with proteins and polyphenols. In contrast, late-boil or dry-hop additions introduce nonisomerized hop material, including vegetal particles, essential oils, and polyphenols, which are absorbed by the trub, and modify both its bitterness and chemical profile. These hop addition timings shape the hop compounds in the trub and directly affect the debittering process discussed later. After boiling, filtration or decantation separates the protein precipitates and other components that did not dissolve, forming tannins and high-molecular-weight proteins complex, known as hot trub. Typically, 0.2 to 0.4 kg of hot trub is generated for every 100 L of wort when separated by whirlpool.^32^ Cross flow microfiltration (with pore size of 0.2 μm, at 10 ± 1 °C, 0.4 bar of transmembrane pressure, and 0.4 m s^–1^ of crossflow velocity) completely removed hot trub and cold trub.^33^

But the quantity varies according to the hopping regime (i.e., hop dosage, hop type, timing of additions, functional purpose, and specific hopping techniques), as hop-rich styles tend to produce significantly larger trub volumes.^32^ Indeed, hot trub yields can vary widely depending on the kettle trub separation method (e.g., whirlpool vs mechanical filtration) because each technique retains different amounts of particulate material, suspended solids and entrained wort, directly affecting the final volume of trub recovered.

Hot trub is a bitter byproduct that limits its direct application in food products. However, its chemical composition has demonstrated the potential to be subjected to extraction and isolation processes of its valuable bioactive compounds.^34^ Thus, hot trub can be used in two primary forms: obtaining fat-rich products or a source of vegetable protein.^35,36^ The wort is then cooled and transferred to the fermentation stage, where *S. cerevisiae* is added.

After fermentation, the yeast is separated by decantation, settling at the bottom of the tank and yielding BSY (approximately 2–4 kg of BSY are generated for every 100 L of beer produced). This biomass is commonly reused as inoculum in subsequent fermentation batches through a repitching process, typically over a limited number of generations. Repitching practices are constrained by yeast vitality and viability losses, contamination risks, and potential flavor drift over successive cycles.^37^ Unfortunately, yeasts can lose their performance after continuous repitching, leading to quality inconsistencies in beer products, especially in terms of alcohol content and generation of volatile compounds, causing off-flavors, impacting their taste.^32,36,37^ Following, the liquid is transferred to maturation, where the stabilization of flavor and aroma compounds occurs, as well as aiding in the clarification of the beer due to the precipitation of yeast and proteins.^32^ Next, beer can be clarified using diatomaceous earth filters, which remove suspended particles and residual trub/yeast sediment. And it can be carbonated, pasteurized, and packaged.^24^ Factors such as the type of yeast used, the filtration process, additives, and the equipment’s cleaning procedures significantly influence the waste generation rate.^25,38^

Brewery wastewater, including streams from equipment cleaning, tank rinsing, and boiler purging are rich in organic matter, nutrients, and chemical compounds.^39^ For every liter of beer produced, between 3 and 10 L of effluent are generated, varying depending on the beer being made.^6,39,40^

### 3.2 Characterization of Solid Waste in Breweries

Solid waste generated by breweries can be repurposed in multiple ways due to its chemical composition. These can be used as a food ingredient, a raw material for microbiological processes or chemical transformations, and in pharmaceuticals, cosmetics, and others.^40^ The composition of the residue can vary greatly depending on the style of beer and the ingredients used. Some examples of the chemical and nutritional composition of solid brewery wastes are presented in Table 1.

**Table 1:** Chemical and Nutritional Composition of Solid Brewery Wastes^a^

	BSG	BSY	Hot trub
Centesimal Composition (g/100 g ww)			
Moisture	76.47–82.6	86.05	86.9
Centesimal Composition (g/100 g dw)			
Ashes (fixed mineral residue)	3.8–4.6	4.9–14	2.00–4.14
Proteins (microkjeldahl)	4.89–26.9	38.82–64.1	26.51–48.8
Lipids (ether extract)	2.67–11	0.62–3.53	3.03–5.23
Crude fibers	4.19	12.22	1.96–2.24
Carbohydrates	45	12.9–21.52	35.36–57.19
Amino Acids (g/100g dw)			
Alanine	0.02	9.29	4.23
Arginine	0.05	6	3.78
Aspartic acid	nd	5.98	5.59
Cysteine	nd	2.19	1.57
Glutamic acid	nd	15	12.2
Asparagine	0.02**	2	nd
Glutamine	0.04***	3.13	nd
Glycine	0.02	3.69	3.08
Histidine	0.02	1.9–2.77	1.73
Isoleucine	0.02	3.23–5.64	3.29
Leucine	0.04	3.51–8.84	7.14
Lysine	0.01	3.16–8.78	3.16
Methionine	0.01–0.04	2.28	1.09
Phenylalanine	0.04	3.01	4.21
Proline	0.07	2.65	5.25
Serine	0.02	4.6	4.03
Threonine	0.02	2.6–6.16	2.86
Tyrosine	0.04–0.10	2.15	2.14
Valine	0.03	3.5–6.20	4.59
Tryptophan	nd	1.1–1.39	0.69
Free Phenolic Compounds (mg/kg dw)			
Ferulic acid	10.56–35.82	0.73–2	1565.5–2908.9
*p*-coumaric acid	-	0.2–0.26	-
Sinapic acid	10.96	nd	-
Caffeic acid	1.33–23.15	nd	-
Syringic acid	13.72–34.33	0.04–0.07	-
Gallic acid	4.2–57.36	0.31–21.3	-
Xanthohumol	0.93	0.56–2.89	Present
Catechin	10.8–116.04	<0.09	-
Vanillin	14.63–27.8	<0.02	-
References	^9,41–53^	^35,44,54–58^	^35,44,59–61^

a ww: wet weight. dw: dry weight; nd: not determined. ** Asparagine + aspartic acid. ***. l-glutamine + l-glutamic acid.

#### 3.2.1 Brewer’s Spent Grain (BSG)

BSG represents 85% of the total brewing waste, comprising the remaining malt and grains after the sugar’s extraction in the mashing process. BSG is a lignocellulosic material whose main constituents are fibers (hemicellulose and cellulose), proteins, and lignin, with appreciable contents of essential amino acids, minerals, phenolic compounds, and lipids. BSG can be regarded as a lignocellulosic material with cellulose (β-(1,4)-linked glucose residues), hemicellulose, lignin, and extractives.^43,44^ The lignin content from BSG varies from 208 to 280 g kg^–1^ (dry matter).^62^ Cellulose and hemicellulose contents found in BSG ranged from 12 to 16.8% and 16.88 to 29.92%, respectively. These values were lower than those reported by Coronado et al.,^62^ who mentioned 26.8 and 37.17% for cellulose and hemicellulose, respectively.

BSG’s fibers can also be classified according to their solubility in the diet. Soluble fiber includes β-glucans, pectic polysaccharides, arabinogalactans, high-branched arabinoxylan, and xyloglucans. Insoluble fibers include lignin, cellulose, low-branched arabinoxylans, xyloglucans, and galactomannans.^45^ Arabinoxylan can act as a prebiotic since microorganisms in the large intestine, such as bifidobacteria and lactobacilli, can ferment this molecule. The breakdown of arabinoxylan produces xylooligosaccharides (XOS) with varying degrees of polymerization, which may be the reason for the prebiotic activity of arabinoxylan.^63^ Enzymatic hydrolysis with a hemicellulase xylanolytic complex yielded more XOS from Pilsen BSG xylan (2.5 mg/mL of total XOS) than from IPA BSG xylan (1.78 mg/mL).^64^

Despite the high arabinan content in BSG, xylan and glucan are the main polysaccharides constituting the hemicellulosic structure. Depending on the evenness of malting, starchy endosperm may also remain.^46,65^ The most abundant monosaccharides in BSG are xylose, glucose, and arabinose, while traces of rhamnose and galactose have also been found.^63^ Jin et al.^53^ reported 8.2% (dry basis) fermentable sugars (maltose, maltotriose, glucose, and fructose) content in BSG from craft breweries. Some authors found 1,89% (wet basis) of the fermentable carbohydrate content in BSG, indicating its fibrous nature due to successive washing steps to recover the brewing wort extract.^46^

The protein content of BSG from craft breweries varies considerably (14.44 to 26.9%). Farcas et al.^66^ reported an average protein content of 24.8% in craft BSGs. Jin et al.^53^ reported an average value of 18.7% in BSG craft samples. The protein content of BSG is higher than that reported for other lignocellulosic materials such as barley straw, oats straw, rice straw, and wheat straw.^41^ The most abundant proteins are hordeins, glutelins (43% and 21.5% of the total extractable protein, respectively), globulins, and albumins (7.5%).^63,67^ The protein content is high since most malt starch is removed during mashing. Furthermore, it has a relatively high content of essential amino acids (approximately 30% of the total protein content), especially lysine, which is often deficient in cereal foods.^63^

Cereals’ lipids are in the endosperm and embryo, providing nutrients and energy for the new, germinating plant. Although the endosperm is almost completely solubilized in mashing, most lipids remain with the spent grains. Farcas et al.^8^ reported that the lipid content of BSG varies between 5.40 and 11% (dry mass). A lipid content of 2.67% in fresh BSG and 6.3% in dry BSG was found (Table 1). Farcas et al.^66^ evaluated the variability of five BSG obtained from different craft breweries. The authors found total lipid content ranging from 5.96% to 7.05% (dry basis). Jin et al.,^53^ studying the chemical composition of BSG malt from microbreweries, found 6.5 and 7.3% of lipids (dry basis). del Río et al.^40^ found a value of 9,2% (dry basis) of lipid in BSG obtained from a brewery resulting from wort prepared from malted barley for ale production. The predominant lipids were triglycerides (67%), followed by a series of free fatty acids (18%), monoglycerides (1.7%), and diglycerides (7.7%) among the lipids in BSG. Linoleic (18:2), oleic (18:1), and palmitic (16:0) acids are the more common fatty acids present in BSG, and low levels of α-linolenic (18:3) and stearic (18:0) acids have also been found.^68^ Linoleic acid (18:2) was the most essential fatty acid, and it was found in triglycerides and diglycerides. The elevated level of this fatty acid is comparable to that of “linoleic acid-rich” vegetable oils, such as grape seed, hemp seed, and wheat germ oils.^8^

Ash content varied from 2.05 to 4.6% (dry basis). Low amounts of ash can be an advantage if the BSG follows a thermochemical conversion pathway, since a high ash content may cause ignition and combustion problems.^62^ Table 2 presents composition of macro and micromineral in 100% barley malt and BSG, according to Meneses et al.,^69^ who studied BSG from a microbrewery, reported similar values.

**Table 2:** Macro and Microminerals in Barley Malt and BSG^a^

	mineral (mg/kg)				
	P	Mg	Ca	Si	S
barley malt	5186	1958	3515	10740	1980
BSG	5333.0	2020.8	1523.2	nd	nd

mineral (mg/kg)					
	Fe	Mn	Zn	Cu	Se
BSG	111.2	47.3	45.6	21.6	3.4

a nd: not determined. Reference: Meneses et al.^69^

BSG also represents a valuable source of phenolic compounds since most of the phenolic compounds in barley grain are contained in the husk, and hydroxycinnamic acids accumulate in the cell walls. Phenolic acids are popular research subjects because of their anticarcinogenic, anti-inflammatory, and antioxidant properties.^65^ de Costa et al.^9^ determined total phenolics in pilsner-type BSG flour from a microbrewery using the Folin Ciocalteau reagent by the colorimetric method. The evaluation was between 211 and 258 mg GAE/100 g of dry BSG.^66,69^ The difference between the BSG total phenols samples could be explained by the type of malt used in the brewing process and the temperatures used for drying and cooking the malt during beer production. Another important parameter that could influence the extraction of phenols from BSG is the technique used, as a lignocellulosic material might entrap the phenolic acid in the cell wall.^66^

BSG is characterized by high water content (76–82.6%). Differences in the chemical composition of BSG, especially from craft breweries, depend on several factors, such as harvest time, cereal variety, malting and mashing conditions, and type of adjuncts added to the brewing process.^5,63^

#### 3.2.2 Brewer’s Spent Yeast (BSY)

Protein content in BSY of 45.6% (dry basis) was found by Mathias et al.^44^ In the study by Vieira et al.,^58^ a value of 64.1% (dry basis) was reported in spent brewer’s yeast biomasses from *S. pastorianus* supplied by a brewery from Portugal. Predominant amino acids found in BSY proteins are leucine, lysine, tyrosine, arginine, cysteine, histidine, isoleucine, methionine, phenylalanine, threonine, tryptophan, and valine.^8^ Therefore, BSY is an excellent source of high-quality protein, with a good potential for applications in the food and dietary supplement industries as a protein-rich ingredient. It was determined that 40% of the total amino acid count was composed of essential amino acids.^58^ A high level (34% dw) of flavor-enhancing amino acids (glutamic acid, aspartic acid, glycine, and alanine) was also determined. A high level of these particular amino acids presents the potential of BSY or BSY extracts as flavor-enhancing ingredients.^6^

BSY polysaccharides are mainly β-glucans, mannoproteins, and glycogen. A high percentage of BSY polysaccharides are insoluble, approximately 83% of total polysaccharide content (dw).^6^ Vieira et al.^58^ determined the levels of B vitamins in brewer’s spent yeast extract using HPLC, and the results were as follows: vitamin B3 (72 mg/100 g), B6 (55.1 mg/100 g), and B9 (3.01 mg/100 g). A similar study by Jacob et al.^70^ determined relatively similar values of an autolyzed industrial BSY product via the same method, differing in the value for vitamin B6 (5.9 mg/100 g dw) from that of Vieira et al.^58^

A value of 5.9% (dry matter) of ashes was found in BSY by Mathias et al.,^44^ lower than that found by Vieira et al.^58^ (14.0%). The variation in the mineral composition of yeast depends on the stage of fermentation/maturation at which it is removed and the number of times it is reused. New and propagative yeasts present more significant phosphate reserves, increasing ash content.^71^ P and Na play a key role in ribosomal protein synthesis and are essential in the cell’s acid–base balance and water retention. Other elements include Ca and Mg, present at 27.11 mg/100 g (dw) and 273.6 mg/100 g (dw), respectively.^58^

#### 3.2.3 Hot Trub

The hot trub, consisting of insoluble high molecular weight proteins, wort, hops, and malt particles, is generated during wort boiling and hops addition.^9^ Their composition can vary according to the type of malt used in the process and brewing process characteristics, being formed by proteins (40–70%), lipids (1–2%), carbohydrates (20–60%), and polyphenols (5–10%).^72^ About 85% of the hop compounds are not solubilized in the wort and can be discarded with hot trub.^9^

The chemical composition of hot trub from some microbreweries is summarized in Table 1. Mathias et al.^44^ found 86.9% moisture content, which results from wort loss in the precipitation of these compounds after boiling, and 2% ash content in hot trub. The main macromolecules in hot trub are carbohydrates, which comprise the sugars formed by the hydrolysis of starch released from the grains during malt boiling. Storage proteins, prolamins and glutelins are responsible for the matrix’s trub insoluble characteristic. Santos and Martins^60^ evaluated the effect of drying and hydrothermal treatments on the thermal, bioactive, functional, and antihypertensive effects of hot trub obtained from a microbrewery. In this study, untreated hot trub was dried in the oven, and a protein content of 42.52% was achieved. After extraction, the protein content increased from 26.51 to 70.34%, presenting many essential amino acids (28.76%, dw), such as valine, phenylalanine, isoleucine, and lysine. However, it is necessary to consider that protein content in hot trub depends on several factors, such as the brewing process, malt, presence of other grains, and the hop used.^72^ The use of hot trub depends on the removal of certain off-flavors. Reduction of trub bitterness can be carried out by aqueous extractions at a high temperature (100 °C for 1 h) in five steps.^35^

## 4 Innovative Applications to Breweries’ Residues

Novel applications for brewers’ wastes have been developed. A patent search was conducted on January 30, 2025 using the Google Patents platform (https://patents.google.com) to identify technological developments involving brewery byproducts. Two independent queries were performed: “brewer’s spent grain” AND “food production” and “brewer’s spent yeast” AND “food production”. The searches returned 15,684 and 6,588 documents, respectively, with no regional, temporal, document-type, or language filters applied in order to maximize recall.

Search results correspond to patent documents indexed by Google Patents, including applications and granted patents, and counts were considered per publication record, without consolidation by patent family. To maximize recall, no regional, temporal, document-type, or language filters were applied.

Geographic contribution by continent was determined based on the applicant’s country of origin as reported in the patent metadata and subsequently grouped by continent. It should be noted that the absence of temporal filters may bias absolute counts and relative contributions toward countries with longer patenting histories; therefore, results are interpreted as indicative of overall technological activity rather than recent innovation trends.

Asia leads globally in patent production for BSG and BSY residues, with China contributing significantly to 70% of Asia’s patents and 41% of total global patents for BSG innovations (Figure 2). For BSY, China holds 56% of the world’s patents and 89% within Asia. Following Asia, Europe and North America dominate in patent production. The European Patent Office, Germany, and Russia are the major contributors in Europe. In North America, the USA is the primary source of patents in this field. Other continents, such as Oceania, South America, and Africa, contribute less to developing innovative waste techniques for brewers. Among these regions, countries like Brazil in South America and Australia in Oceania show potential for increased contributions.

**Figure 2:**
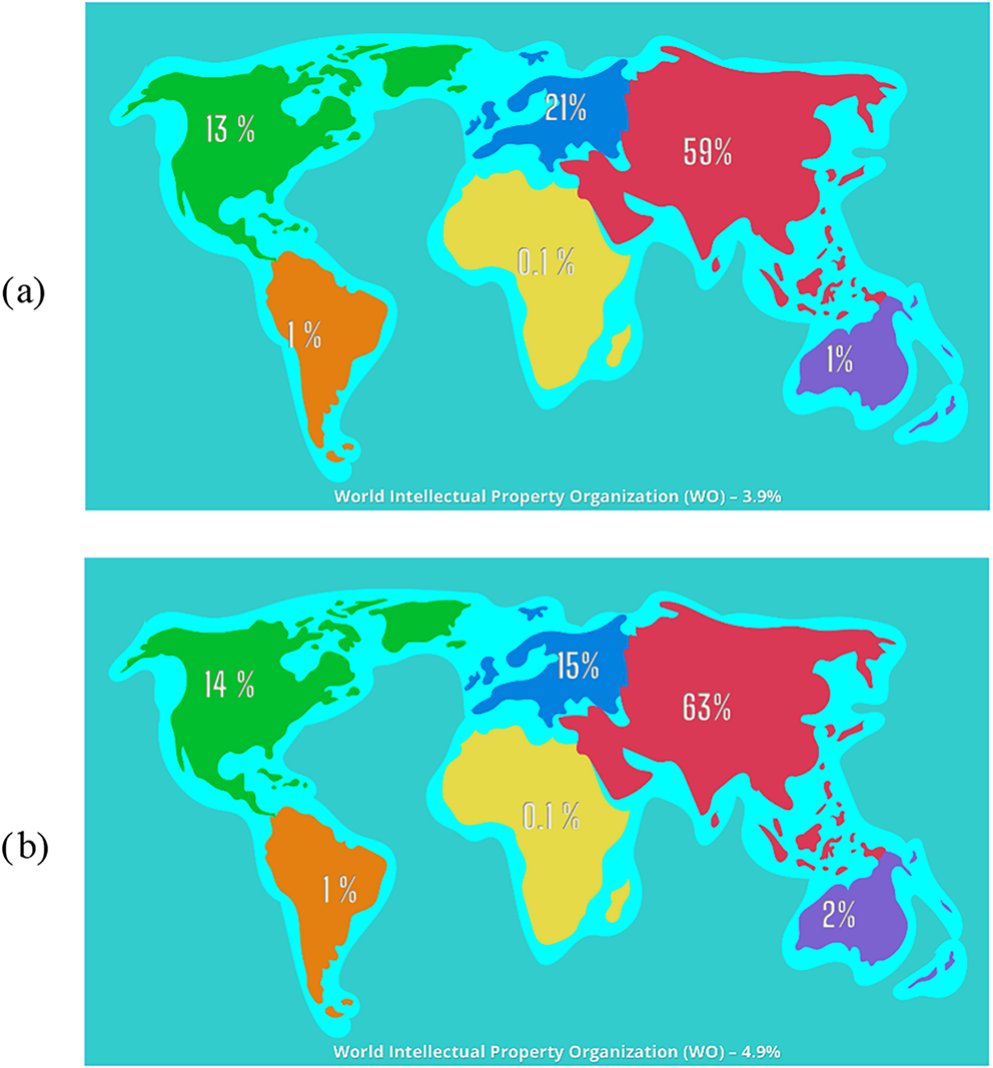
Continental contributions for innovations in (a) BSG and (b) BSY waste management. IPC/CPC classes assigned by the platform were retained, and duplicate filings were consolidated using INPADOC family grouping. The data set was then normalized in Excel to remove remaining duplicates, standardize country codes, and calculate patent counts by country. The graphic was created by the authors using Canva (www.canva.com), based on data retrieved from Google Patents (https://patents.google.com/).

Given the significant global interest in developing innovative applications for brewers’ waste, particularly in regions like Asia, Europe, and North America, there is a growing recognition of the need for sustainable practices within the brewing industry.

### 4.1 Innovative Applications to BSG

There is a broad range of alternatives to recycle BSG. For example, BSG can be transformed using fermentation, enzymatic treatments, and microbial processes to produce biomolecules, biofuels (e.g., biogas, bioethanol), biodegradable plastics, and other value-added products such as biopolymers and prebiotics. Based on the principle of sustainable food systems, the BSG has been widely used as livestock feed, particularly for cattle, pigs, poultry, and fish; due to its high fiber and protein content, it is a cost-effective supplement that reduces waste.^7^

Another example of BSG use is as an activated carbon. BSG subjected to low-temperature carbonization yielded semicoke, which can be subsequently processed into activated carbon. BSG activated carbon has proven to be a cost-effective and efficient sorbent for use in water treatment plants, particularly those located near breweries.^73^

BSG can be converted into biochar through pyrolysis followed by CO_2_ activation. Franciski et al.^74^ produced both biochar and activated biochar, which were characterized and tested as adsorbents for the removal of methylene blue from aqueous solutions. Activated biochar exhibited a typical mesoporous structure with a surface area of approximately 80 m^2^/g, while the maximum adsorption capacity of BMB reached 161 mg/g.

Microwave-assisted hydrothermal carbonization was applied to BSG to produce hydrochar.^75^ Temperature (180–250 °C) and reaction time (0–2 h) significantly affected yield and properties, enabling up to 47% conversion of BSG into high-energy hydrochar (32 MJ/kg), which was mesoporous, hydrophilic, and rough, with surface cavities and oxygenated groups that enhance its aromatic nature and suitability for bioadsorption.

Fernandes et al.^76^ used *Aspergillus ibericus*, a nonmycotoxin-producing fungus, to ferment BSG in a solid-state fermentation (SSF) process to improve protein and reduce fiber content. Additionally, SSF produced a crude enzyme extract rich in cellulase and xylanase, which was used to hydrolyze BSG, obtaining a fish feed supplement. The results showed that supplementing European seabass (*Dicentrarchus labrax*) feed with this enzymatic extract improved their growth and feed efficiency, showing that after biological treatment, enhanced nutritional properties on BSG were obtained to make it a sustainable feed additive for aquaculture.

Gil-Castell et al.^77^ investigated using BSG as a biofuel for energy recovery. The study examined its decomposition during combustion, highlighting two main stages of thermal breakdown involving hemicellulose, cellulose, and lignin. BSG showed promising energy potential with low ash content (1.7–5.4%) and a high calorific value (17.8–19.1 MJ/kg), making it a feasible renewable energy source for incineration processes.^77^

BSG has also been used to produce bioethanol through a one-pot process that combines pretreatment, saccharification, and fermentation in a single bioreactor, eliminating the solids removal operation. BSG was pretreated with dilute acid at 15% (w/w) solids for 1–1.5 h, generating a hydrolysate containing approximately 60 g/L fermentable sugars, which subsequently were fermented using a genetically engineered *Escherichia coli* MS04 strain, producing 29.5 g/L of ethanol after 30 h. The method achieved a bioethanol yield of 251 L per ton of BSG, showcasing its strong potential for sustainable, energy-efficient bioethanol production.^78^ This study is essential for developing second-generation biofuels, highlighting how to exploit industrial waste while minimizing energy consumption in bioethanol production.

Ludka et al.^79^ explored how BSG can be used as a sustainable source of antioxidants in starch-based materials. The study aimed to develop films from starch and poly­(vinyl alcohol) (PVA), incorporating BSG extract. The application of BSG extract resulted in films with antioxidant activity without changes in mechanical, structural, and barrier properties.

Similarly, BSG can contribute to the protection of probiotic cells. BSG flour has improved probiotic strains’ recovery in fermented milk, as reported by Battistini et al.^80^ The study also evaluated probiotic exposure to simulated gastrointestinal conditions, including extreme pH and bile salts, and showed that BSG flour increased strain viability while providing a supportive matrix for survival. These effects were attributed to the nutrients and fiber present in BSG, which act as substrates that enhance resistance to adverse conditions. In addition, the incorporation of BSG flour influenced the sensory profile of fermented dairy products, typically imparting a darker color and subtle cereal notes.

### 4.2 Innovative Applications to BSY

Discarded yeasts are rich in nutrients, particularly proteins, vitamins, and minerals, and they also contain, in lower amounts, bioactive compounds such as polyphenols. Yeast biomass typically contains 8–11% (dry mass) nucleic acids, compared to approximately 0.2% in meat products.^81^ High nucleic acid intake may lead to increased uric acid levels in the bloodstream, which is associated with hyperuricemia and uric acid deposition in joints. Consequently, elevated nucleic acid content can limit the direct use of yeast biomass in human foods unless appropriate processing is applied. Reduction of RNA content can be achieved through heat-induced autolysis followed by enzymatic RNase treatment, which mitigates these health concerns and expands the potential for food applications.

Therefore, BSY is mainly used in animal feed.^6,71^ Heringer et al.^71^ highlight BSY’s nutritional value and bioactive potential, particularly in producing lasiodiplodan, a β-glucan polymer with therapeutic properties and single-cell protein, which can serve as a sustainable protein source. The research focused on optimizing the fermentation process to enhance (1 → 6)-β-d-glucan production from BSY while generating high-quality single-cell protein. The study evaluated various parameters affecting the yield and quality of the polysaccharide and protein, aiming to develop a cost-effective and environmentally friendly process.

Protein hydrolysates derived from BSY have been demonstrated to have excellent technological properties, such as effective emulsifiers and carrier materials for microencapsulating edible oils, specifically sunflower oil.^82^ Other possibilities regarding the use of BSY include the extraction of β-glucan and other cell wall polysaccharides, protein extraction, bioactive peptides, use as a food ingredient (meat substitute, bakery products, and savory snacks), functional food additive, as well as nonfood applications.^6,56^ Therefore, BSY has the potential to be applied in a wide range of ways, such as recycling materials and improving the production chain.

### 4.3 Innovative Applications to Hot Trub

Trub is rich in proteins and fibers but contains bitter compounds that must be removed for human consumption. This waste is commonly used in animal feed, especially for cattle and pigs. However, alternatives for their use in the human diet have been investigated. Saraiva et al.^83^ explored the extraction of debittered trub (DT) as an ingredient in pasta. The research evaluated the effects of adding DT at different levels (5, 10, and 15%) on the pasta’s technological, chemical, and sensory qualities. The results show that up to 10% of trub does not compromise the quality or sensory acceptance of the pasta. Additionally, nutritional improvements, such as increased protein digestibility and reduced glucose release during starch digestion, could benefit metabolic health. Thus, debittered trub offers a sustainable and nutritious solution for enriching foods like pasta and reducing waste in the brewing industry.

Santos and Martins^60^ evaluated trubs’ functional and thermal properties, focusing on their potential health benefits, including antihypertensive effects. The research highlighted that hot trub is rich in bioactive compounds and proteins that should offer functional properties in food applications. Its protein content shows promise for developing alternative protein sources, especially in sustainable food systems. The study also examines the trub’s antioxidant activity and its potential to inhibit hypertension-associated enzymes, suggesting possible cardiovascular health benefits. Moreover, the thermal analysis confirms that hot trub proteins have good stability, making them suitable for various food processing methods. This study positions hot trub as a valuable ingredient that could reduce food waste while enhancing the nutritional profile of foods. Trub has also been used for the extraction of bioactive phenolic compounds,^84^ the extraction of protein (highly soluble proteins),^85^ and for recovering bitter compounds.^86^

### 4.4 Brewery Wastewater Management

In addition to solid wastes, wastewater from breweries also raises environmental concerns. Commonly, it is estimated that brewing one liter of beer requires nearly ten liters of water, primarily for brewing, rinsing, and cooling. Afterward, this water must be either treated for reuse or disposed of safely, which is often expensive and challenging for most breweries.^87^ Simate et al.^87^ highlight that beer production results in large volumes of wastewater containing a high organic load and nutrients, making it difficult to achieve effective treatment for reuse. The authors describe a range of physical, chemical, and biological treatment processes, such as sedimentation, filtration, activated sludge, anaerobic digestion, and membrane reactors, and emphasize the importance of reusing treated brewery wastewater, noting its potential applications in irrigation, industrial cleaning, and various brewing operations.

Verhuelsdonk et al.^88^ performed a study on a pilot modular system for brewery wastewater reuse comprising flotation, membrane bioreactors (MBR), ultrafiltration, and reverse osmosis, achieved 93.6% chemical oxygen demand (COD) removal and produced process water of drinking quality with a 63% yield. Economic analysis using a probabilistic simulation tool (75,000 iterations) showed that full-scale reuse is viable in 77.2% of scenarios, with outcomes most sensitive to wastewater disposal costs.

In a study evaluating the wastewater treatment system at the Heineken Brewery in Ethiopia, the plant achieved 97.2% removal of COD and biological oxygen demand (BOD_5_) and 95.7% of total suspended solids (TSS), with all values falling below discharge limits. Total nitrogen (TN), total phosphorus (TP) removal rates were 49.4 and 57.6%, indicating that there is still room for process improvements.^89^

Wastewater contains recoverable chemical energy, which can be harnessed through microbial fuel cells (MFCs) using electroactive bacteria. An investigation showed that autochthonous microorganisms isolated from brewery waste sludge enabled simultaneous wastewater treatment and energy recovery in MFCs, achieving power densities of 0.8 W/m^3^ with synthetic wastewater and 0.35 W/m^3^ with real brewery effluent, while removing 79–83% of COD.^90^

## 5 Sustainability in Microbreweries: Challenges and Perspectives within the Circular Economy

Brewery byproducts are generally recovered and applied as soil fertilizer or human and animal feed. BSG is usually applied as a healthy food ingredient alternative. However, BSY and trubs can negatively affect the sensorial properties of foods when directly used as a substitute for raw ingredients. Although these brewing subproducts are rich in proteins, fatty acids, dietary fiber, and bioactive compounds, their unique flavor derived from tannins can negatively impact food flavor when directly applied to food products. Thus, technologies have been developed for the previous extraction, isolation, and purification of valuable compounds.^34,35,81,91^

The sustainable management of brewing waste presents a promising opportunity to address environmental challenges while unlocking economic and technological potential.^37^ By integrating innovative approaches such as fermentation,^29,92^ enzymatic treatments,^28,93^ and biofuel production,^77^ brewery byproducts like BSG and BSY can be transformed into valuable resources.^67,85,94^ These efforts reduce waste and contribute to circular economy models prioritizing resource efficiency.

However, despite the promising trends for utilizing these residues, significant gaps remain between research and real-world application. Brewer’s spent grain (BSG) is characterized by high moisture content (typically 75–80%), which imposes substantial logistical and energetic constraints, particularly in microbreweries.

Based on typical BSG generation rates and bulk density, wet BSG may require approximately 2–3 m^2^ of temporary storage per 1000 L of beer produced, depending on residence time and handling practices. Moreover, energy demands associated with stabilization or drying processes can be considerable, as moisture reduction from ∼80% to levels suitable for storage or valorization requires substantial thermal input. If wet BSG is not processed immediately, chilled storage is often necessary to prevent microbial spoilage. These logistical and energy constraints help explain the persistent gap between laboratory-scale valorization strategies and industrial implementation, highlighting the need for scalable biotechnological solutions and coordinated waste management strategies.

In general, large breweries are generally more sustainable than microbreweries (lower environmental impact per liter of beer) because they have more rigorous production control and significant investment in various areas, including waste management. Usually, microbreweries do not have a wastewater treatment plant (WWTP) and tend to hire third-party companies to collect effluents to discharge in municipal plants, which can lead to system overload.^22,95^ Another issue microbreweries face is the high initial investment required for equipment that enables efficient waste processing, such as dryers, freezers, or fermentation systems.^95^ Additionally, logistical costs associated with the transport and storage of BSG and BSY are high due to their high moisture content, which reduces the shelf life of the residues, increasing handling expenses. Also, the physical space limitation, as many microbreweries operate in small facilities, makes it challenging to install the necessary equipment.^96^ Despite these difficulties, there are viable paths to make microbreweries more sustainable, and some are constantly seeking ways to become more sustainable.^97^ Within a circular economy, which aims to maximize resource use and minimize waste, they can transform byproducts into value-added products, reducing environmental impact.^97^

A promising strategy consists of establishing partnerships with other companies, allowing waste to be repurposed into products such as animal feed, fertilizers, or bioenergy. More accessible technologies, such as solar drying or small-scale fermentation, can be adapted to extend the shelf life of byproducts without requiring high investments.^98^ Furthermore, engaging employees, suppliers, and consumers in a circular economy is essential to promoting sustainable practices throughout the production chain. Another solution involves internally repurposing waste, such as creating new products, for example, snacks or cookies made from BSG, which adds value to the brand while reducing waste.^99–101^ However, this approach requires caution due to potential allergen and labeling implications, as well as the need to control shelf life and water activity to ensure product safety and stability. In agriculture, the use of brewers’ wastes as fertilizer is frequently mentioned. Studies regarding the impact of these residues on soil quality and plant growth are essential, as they offer sustainable alternatives for agriculture.^102^ Lastly, reusing wastewater through simple treatments like lagoons, biological filters, or wetlands can be an efficient alternative to treat wastewater, minimize water consumption, and promote reuse.^87,103^

Figure 3 illustrates how brewery residues can be integrated into a bioeconomy and biorefinery framework. The numbered labels denote the main residue streams: (1) BSG, (2) BSY, (3) hot trub, and (4) wastewater. Each stream corresponds to specific valorization pathways, including bioethanol, biochar, hidrochar, and human food applications from BSG (1), protein hydrolysates, biomolecules and feed ingredients from BSY (2), protein hydrolysates and biomolecules from hot trub (3), and microalgae cultivation or bioenergy recovery from wastewater (4). This structure improves the connection between conceptual elements and unit operations while reinforcing the role of residue valorization in circular bioeconomy strategies.

**Figure 3:**
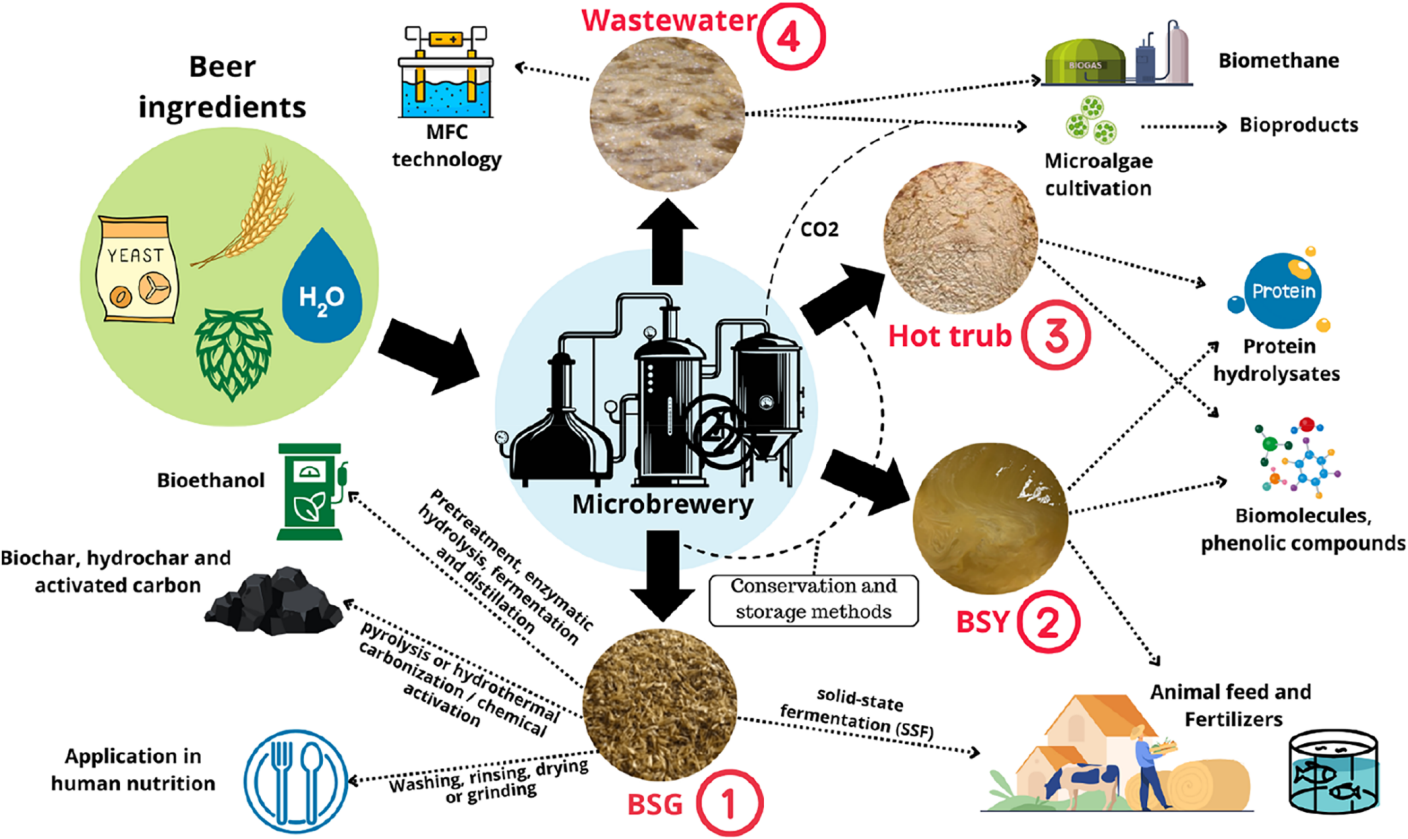
Proposed cycle for the circular economy in brewers or microbreweries.

One of the main challenges of using BSG is the high moisture content, which limits storage and transport. The economic feasibility of transforming this waste on a large scale is also a challenge due to costs related to energy, water usage, and processing technology.^104^ Therefore, Chattaraj et al.^104^ suggest integrating brewery waste recycling into a circular economy framework to reduce environmental impact while creating sustainable, profitable uses, developing more efficient biotechnological methods for waste valorization, and encouraging industries to reduce waste generation. If brewery waste is to become a raw material in other processes, it must maintain its quality and integrity from a physical, chemical, and biological point of view. Applying correct conservation techniques requires knowledge of the degradation processes of the compounds in question.^12^

Regarding physical contamination, waste can contain fragments of foreign compounds such as metals, plastics, wood, glass, hair, insects, nails, and other objects or pieces of objects present in the production process.^105^ Recommended practices to minimize physical contamination in brewery residues include: (a) using closed and well-maintained bins or containers for handling and storage; (b) implementing routine pest control to prevent insect or rodent intrusion; (c) applying sieves or magnetic traps when relevant to remove metal fragments or other particulates; (d) maintaining clean processing areas to avoid contamination from structural materials such as wood, glass, or plastics; and (e) ensuring the use of appropriate personal protective equipment (gloves, masks, caps) during handling.^106^

Chemical contamination can occur in two main ways. The first is by process components generated during or after fermentation by alternative yeast metabolic routes, contaminating microorganisms, or by chemical degradation of the residue through exposure to oxygen, light, excess humidity, high temperatures, and other factors. To avoid these alterations, strict quality control of the fermentation process is necessary, guaranteeing the formation of the compounds of interest and avoiding contamination. In addition, the correct handling and storage of postfermentation waste should be ensured, and new batches should not be mixed with already contaminated ones.^105^ This prevents the fermentation process from continuing in the waste since the lack of nutrients will stimulate other metabolic routes in the yeast, generating contaminating products or promoting the development of contaminating microorganisms, thus promoting microbiological contamination.^107^

Another common form of chemical contamination is by chemical products in the factory, whether ingredients in the product formulation or, more importantly, products used to clean, sanitize equipment and those from the cooling system. Contamination may also occur directly by adding chemical products when a product is mistakenly added to a formulation.^105^ However, the most common way is accidental contamination, when residues of chemical products or their vapors are present in storage containers and equipment due to deficiencies in the rinsing stages. To avoid this contamination, the correct labeling and storage of chemical products, usage protocols, and continuous operational staff training can be adequate.^108^ A notable case of severe chemical contamination in the brewing sector was the Backer incident in Brazil in 2020, where a leak of diethylene glycol and monoethylene glycol from the cooling system infiltrated in the beer tank. This event clearly illustrates how failures in equipment integrity can also lead to widespread contamination across all byproduct streams.^109^ The primary chemical pollutants detected during beer manufacturing are acrylamide, aliphatic chlorinated hydrocarbons, biogenic amines, bisphenols, carbonyls, furans and derivatives, heavy metals, microplastics, mycotoxins, nitrosamines and ATNC, pesticides, phythalates, polychlorinated biphenyls (PCBs), polycyclic aromatic hydrocarbons (PAHs), and trialomethanes. Table 3 summarizes the main chemical contaminants, their likely sources, and the byproduct streams where they tend to concentrate.

**Table 3:** Main Chemical Contaminants in the Brewing Chain

chemical contaminant	likely source	byproduct stream where it concentrates
PAHs (polycyclic aromatic hydrocarbons)	Kettle caramelization, high-temperature heating surfaces.	Hot trub, kettle deposits
Pesticides	Residues from malted barley and hops.	BSG, hot trub
Mycotoxins	Contaminated grains before malting.	BSG
Heavy metals	Equipment corrosion, piping, water quality.	Wastewater, hot trub
Acrylamide	Maillard reactions during kilning and mashing	Hot trub
Nitrosamines/ATNC	Malt kilning, reactions during boiling.	Hot trub, beer
Biogenic amines	Microbial contamination or spoilage.	BSY, wastewater
Bisphenols, phthalates	Plastics, hoses, seals, packaging.	Wastewater, trace levels in BSG
Polychlorinated biphenyls (PCBs)	Legacy contamination in water or industrial areas.	Wastewater
Trihalomethanes	Chlorinated water disinfectants.	Wastewater
Carbonyls, furan derivatives	Thermal processing, caramelization.	Hot trub
Microplastics	Water sources, equipment abrasion.	Wastewater
Dietylene glycol (DEG) and monoethylene glycol (MEG)	Accidental contamination from improper use or leakage of cooling or other system fluids.	Beer and all downstream streams, BSY

Additionally, pollutants may originate from the soil or the water used, which constitute approximately the primary ingredients. The brewing process can introduce various chemical pollutants, including mycotoxins, nitrosamines, heavy metals, biogenic amines, phthalates, bisphenols, pesticides, and acrylamide. In recent studies, microplastics have also been identified, along with trace amounts of polychlorinated biphenyls, trihalomethanes, carbonyls, furan derivatives, and hydrocarbons, both polycyclic aromatic and aliphatic chlorinated.^110,111^

Biological contamination usually occurs through the presence of undesirable microorganisms (spoilers or pathogens), insects, and rodents.^112,113^ To prevent contamination by insects and rodents, a pest management plan is essential, as is raising awareness among handlers of the importance of keeping doors and windows closed or protected by screens during handling. Keep product storage containers closed when in use and protected or inverted when empty, storing waste in an appropriate area within the production or storage environment, avoiding storing it outside and with access to people who are not involved in the process. Preventing microbiological contamination begins when the raw materials are received, continues during fermentation, and requires special attention at the end of the fermentation process and during storage. The hygiene of the environment, equipment, and operators, as well as the correct process execution, promote the development of *S. cerevisiae* to the detriment of other contaminants.^108^ However, after the process, the exposure of the waste to the air, the lack of care in handling and storage, as well as the lack of water and nutrients, combined with the lack of temperature control, can encourage the development of other yeasts, fungi, and bacteria with potential deteriorating or pathogenic action.

However, the degradation of brewing waste cannot be prevented by prevention alone. Action is needed to inhibit the organic compounds’ natural metabolism, leading to deterioration. Among the most common conservation methods are refrigeration, pasteurization, sterilization, dehydration, and chemical additives.^114^ The conservation method application maintains the waste’s quality for extended periods. This allows for greater flexibility and efficiency in planning the transportation to the processing site. It can also add value to the waste since maintaining its quality is essential if it is to be used in processes to obtain products with greater added value.^115^

## 6 Trends and Applications of BSG to Food Formulations

BSG is emerging as an ingredient in a variety of food products such as bread, cookies, baked snacks, pasta, noodles, muffins, yogurt, plant-based yogurt, ready-to-drink, confectionery, sausage, burgers, and mayonnaise^27^ (Figure 4). It contains several nutrients, such as bioactive dietary fibers, polyphenolic compounds, proteins, amino acids, fatty acids, and minerals, which are responsible for these health benefits. In general, BSG improves the nutritional value of the end products. However, it also impacts the technological processing, mechanical, and physicochemical properties of the final product, including visual appearances and final acceptability^116^

**Figure 4:**
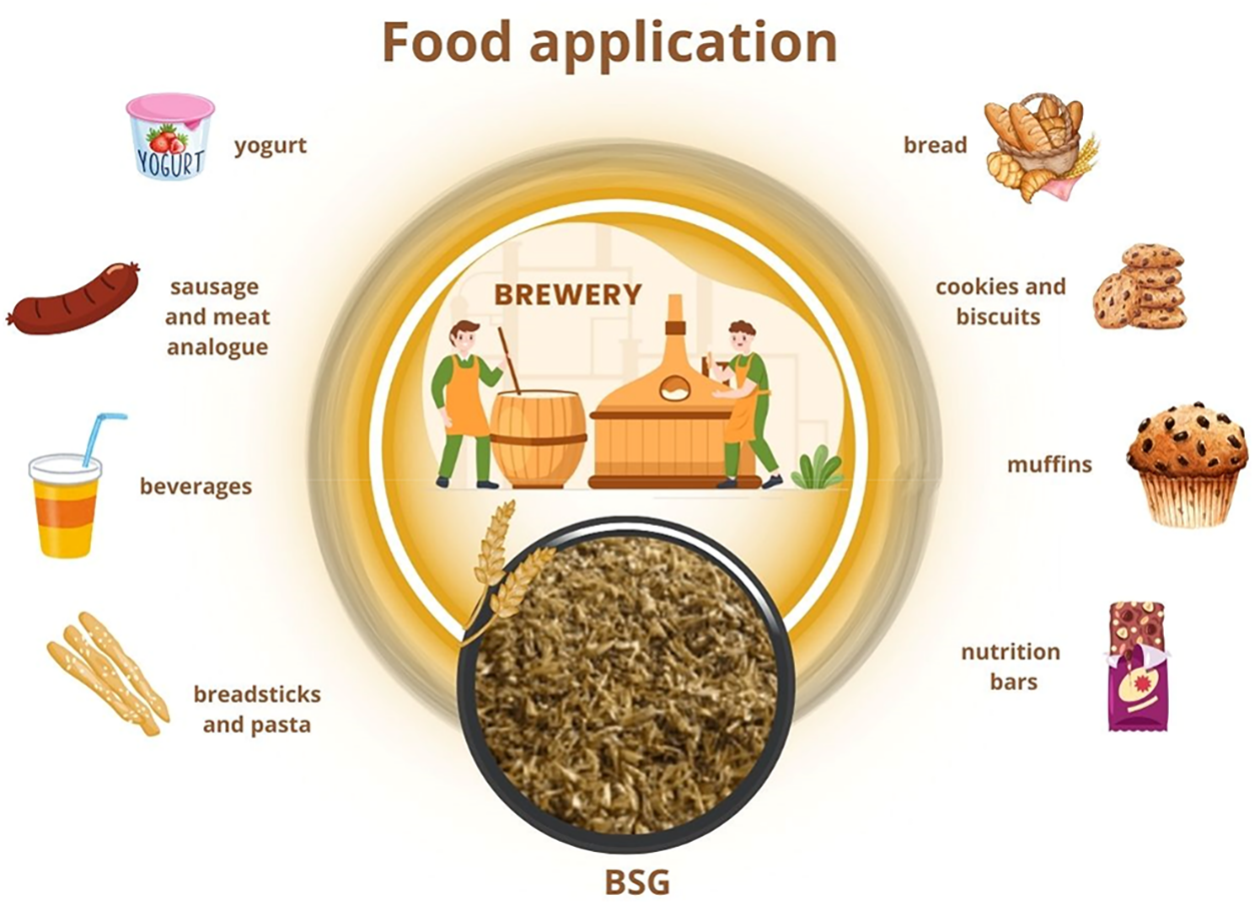
Food applications to BSG.

Many researchers evaluated the technological aspects of BSG as an ingredient in food recipes, generally as a flour substitute, with optimal incorporations rates (%) varying by products: bread, pasta, muffins, cookies, and cereal snacks. These applications have been the subject of sensorial studies. The sensory characteristics of food products are essential. The BSG application in food processing has positive health effects. However, from a consumer’s view, sensory properties are the second most crucial factor.^99,117^ Consumers have been more interested in new healthy recipes. Therefore, developing high-nutrition and low-cost food alternatives by reusing byproducts is a current challenge.^36^

Spent grain is usually pretreated to make its components more accessible, through the decreasing particle size and opening of cell wall structure, consequently improving digestibility.^36^ Thus, BSG is dried and milled to achieve a moisture of 5% and particle size 125–840 μm to obtain BSG flour.^67^ The time spent to dry varies from 72 h at 40 °C, usually 24 h at 60 °C in a conventional oven, and up to 1h in vacuum-dried at 80 °C.

The extrusion process was studied to produce cereal snacks. Although the process requires equipment investment, it positively affects the interest in spent grain for some products.^36^ Extrusion is a thermo-mechanical process that disrupts the cell matrix, obtaining more soluble fragments with a higher content of soluble dietary fibers. The method also generates a microbiologically safe product.^118^ Other approaches involve the extraction of compounds from BSG. Extraction yields fiber and/or protein; considering nutritional and health-related compounds in BSG, extracts are applied to produce emulsions and beverages such as vegetal yogurts.^67,119^

BSG application in foods shows sensory limitations, thus the adequate addition level as a substitutive ingredient is between 2.5 and 30%, depending on the products and treatments. Sensory limits of BSG addition range to 10–15% for bread and cookies, and 5–10% for snacks and pasta products (Table 4).

**Table 4:** Application of BSG in Food Products

product	process	results	references
Bread	Barley BSG and barley-buckwheat BSG, used to replace wheat flour in bread formulation (0, 10, and 20%).	The partial replacement of wheat flour reduced gluten yield, with lower sedimentation values and dough stability, along with increased dough softening. The starch-enzymatic system was also affected, resulting in a lower falling number and higher peak paste viscosity. The addition of brewers’ spent grain (BSG) increased bread yield but decreased loaf volume. On the other hand, the nutritional properties improved, with higher levels of protein, dietary fiber, fat, and ash. A 10% substitute of wheat flour with BSG showed the best results. Moreover, bread made with BBSG + B achieved higher scores than those made with BBSG alone.	^120^
Bread	Obtention of bread manufactured through wheat, maize, and BSG flour.	Sensory characteristics such as color, taste, flavor, texture, and overall acceptability declined noticeably as the proportions of BSG and maize flour increased. Optimization of the bread formulation revealed that the most preferred combination consisted of 65% wheat flour, 20% maize flour, and 15% BSG flour.	^121^
Bread	Enzymatically treated BSG, commercial amylase, xylanase, and endoglucanase were incorporated into bread formulations at up to 30%.	The addition of enzymes improve the bread’s texture, increased loaf volume, and extended its shelf life.	^93^
Bread	Sourdough fermentation of BSG bread using *Lactobacillus brevis*, *Lactobacillus plantarum*, and *S. cerevisiae* strains.	Using a sourdough starter culture containing *L. brevis*, *L. plantarum*, and *S. cerevisiae* positively influenced the properties of BSG-enriched bread. This was evidenced by a reduction of over 30% in phytic acid content and an increase in antioxidant activity of up to 36%, compared to bread made with unfermented BSG.	^92^
Biscuits	BSG is added in biscuit recipes as BSG flour after drying until 10% moisture and milled.	Adding 10% BSG to the biscuits imparted a pleasant wheat aroma and a chocolate-like appearance.	^122^
Biscuits	BSG-fortified biscuits were tested by mixing wheat flour, oats, and BSG flour.	Ingredient replacements affected the sensory characteristics and consumers’ preferences of the BSG-fortified biscuits. Different clusters of young consumers had different preferences and different textural perceptions of these biscuits.	^123^
Cookies	BSG with particle sizes ranging from medium to coarse (∼200–850 μm) was incorporated as a supplementary ingredient in cookie preparation.	Cookies enriched with medium and coarse BSG particle sizes were superior to those made with finely ground BSG. Overall sensory evaluation showed lower substitution levels using medium and coarse BSG, which were rated as equal to or better than the control samples. Panelists accepted the cookies with BSG additions up to 15%.	^11^
Sheets for lasagna	Lasagna sheets were crafted using BSG flour, enriched with fiber from fresh eggs. The BSG underwent vacuum drying at 60 °C for 48 h before being finely milled to a size below 500 μm. The egg component was incorporated in liquid form, utilizing pasteurized whole egg and egg white powder (EWP).	Pasta quality optimization using the desirability function showed that BSG and egg white powder (EWP) can be effectively utilized to develop a fresh egg pasta classified as a “source of fiber.” The findings demonstrated that leveraging the structural properties of EWP enables the incorporation of BSG into a value-added pasta product with enhanced nutritional claims. Although the optimized formulation included a modest amount of BSG (6.2%), this represents a significant outcome for breweries, as BSG valorization contributes to enhancing the sustainability of the brewing industry.	^124^
Grits snacks	BSG was added in the percentages of 5,10, and 15% of the grits extruded snacks.	Extrusion and the incorporation of BSG significantly influenced the protein content in corn-based snacks. The extrusion process led to a decrease in insoluble fiber and an increase in soluble fiber. Adding BSG reduced the expansion ratio and fracturability of the extrudates, while increasing bulk density and hardness. However, the inclusion of 1% pectin effectively addressed the issue of limited expansion in BSG-containing snacks. Among the formulations, those with 5% BSG achieved the highest scores across all evaluated parameters, including external appearance (uniformity, color), structure (porosity, crispness), texture (chewiness), as well as aroma and flavor, when compared to the control corn grits extrudate.	^125^
Breadsticks	Breadsticks were formulated with 25% and 35% of BSG.	BSG notably enhanced the protein levels in the snacks, and incorporating 15% BSG led to more than a 2-fold increase in dietary fiber content within the samples.	^126^
Crispy-slices baked snacks	BSG flour (moisture 5.6%) was added in different percentages in crispy-slice baked snack formulations.	The 10% BSG formulation achieved the highest average ratings across all quality attributes, including texture, flavor, aroma, appearance, and overall acceptability. However, as BSG concentration increased, a decline in acceptability was noted, with the 25% BSG samples being the least favored.	^101^
Muffins	BSG was used for the fortification of muffins.	Muffins fortified with 15% and 20% BSG exhibited enhanced fat, protein, and dietary fiber content, and increased bioactive compounds. However, the 15% BSG formulation had lower batter viscosity and maintained a brighter muffin color than the 20% BSG version, potentially leading to higher consumer acceptance. As a result, the 15% BSG-fortified sample was selected for sensory evaluation. The study aimed to introduce value-added products to consumers and assess their interest.	^127^
Meat analogue product	BSG was incorporated into soy protein-based high-moisture meat analogues (HMMAs) to enhance their nutritional profile and functional properties.	Incorporating 15% BSG enhanced texturization and reduced the hardness of HMMAs. However, higher levels of BSG negatively affected the formation of a fibrous structure, which is essential for replicating the mouthfeel of animal-based products. The addition of BSG in soy protein-based meat analogs demonstrated strong potential for expanding the variety of plant-based meat products in a more sustainable and nutritionally beneficial way.	^10^
Sausage	Hybrid sausages were formulated by combining broccoli, BSG, and insect flours from *Tenebrio molitor* (IF).	Integrating alternative protein sources is a promising strategy for reducing animal protein in meat formulations. The blend with 22% broccoli, 3% BSG, and 10% IF exhibited juiciness and aroma comparable to the commercial sample, while also outperforming it in appearance.	^128^
Sausage	Pork back fat was replaced with BSG pre-emulsion in chicken sausages.	A pre-emulsion was created using 15% BSG dietary fiber extracts, and sausages were formulated by substituting 20, 25, and 30% of pork back fat with BSG pre-emulsion. This substitution improved the reduced-fat chicken sausage’s hardness, gumminess, and chewiness. Fat replacement with 20–25% BSG pre-emulsion enhanced the texture and quality of these sausages.	^129^
Coconut extract-based yogurt	BSG flour (BSGF) and protein extracts (BSGPs) were incorporated into water-soluble coconut extract (WSCE) based yogurt alternatives.	BSGF and BSGPs enhanced mechanical properties, viscosity, and flow behavior modifications, highlighting their capacity to sustain texture and gel formation. After 14 days of storage, stability in flow behavior, syneresis, and mechanical properties was maintained.	^130^
Yogurt milk	BSG was added in different concentrations to the yogurt formulation.	Replacing 5%–10% of BSG in yogurt enhanced its overall quality, optimizing acidity, rheological behavior, and LAB growth. While incorporating 15%–20% BSG resulted in similar acidity and LAB growth with minimal syneresis, it negatively impacted the yogurt’s flow performance.	^131^
Novel nutritional beverage for human health	A novel beverage was created through the submerged fermentation of BSG utilizing *Bacillus subtilis* WX-17, without adding supplementary components. This approach highlights the potential of BSG fermentation to enhance product functionality and nutritional benefits naturally.	The fermentation products were extracted in the liquid phase, yielding a promising nutritional beverage containing *B. subtilis* WX-17, which remained viable for 6 weeks with a final cell count of 9.86 log CFU/mL. This beverage demonstrated increased amino acid levels, phenolic content, and antioxidant activity. The combination of enhanced nutritional properties and viable B. subtilis supports its potential as a novel dietary beverage beneficial to human health.	^132^

Some examples of products obtained by replacing raw ingredients with BSG are shown in Table 4. Also, the use of BSG in such products is discussed in the following topics.

### 6.1 Application in Baking Processes

The BSG flour has been tested in bread formulations to replace wheat flour. Usually, 10% of BSG flour, replacing wheat flour in bread recipes, is well accepted by consumers. BSG inclusion in bread has been challenging because of its high impact on dough rheology and bread quality characteristics. Commercial enzymes have improved BSG flour in previous LAB fermentation and blended with other flours such as rye, corn, and other cereal flours.

The partial substitution of wheat flour (WF) with barley BSG and barley-buckwheat BSG was assessed for its effects on dough quality and bread characteristics, including nutritional value. Breads containing both types of BSG outperformed traditional wheat bread in nutritional value. As the proportion of BSGs in the flour blend increased, protein, dietary fiber, fats, and ash levels rose significantly, while energy value declined. BBSG + B proved to be a more effective additive than BBSG alone. In the organoleptic evaluation, breads with 10% BBSG and BBSG + B exhibited similarities to the control wheat bread, with the BBSG + B formulation receiving higher scores than BBSG-containing bread.^120^

Incorporating flour from various cereals can enhance the proportion of BSG in bread formulations. A recipe combining wheat flour, maize flour, and BSG flour was evaluated, revealing that sensory attributes, such as color, taste, flavor, texture, and overall acceptability, diminished with increasing BSG and maize content. Sixteen experimental trials were designed using Design Expert software and a panel of 50 consumers, aged 20 to 45 years, was randomly selected to perform the sensory evaluation of bread samples. For each sample, participants assessed their preference for the attributes using a 9-point hedonic scale (1 = dislike extremely; 9 = like extremely). Samples were considered acceptable when all sensory attributes achieved scores of 6.00 or higher (like somewhat). Optimization of the recipe determined that the most favorable bread composition consisted of 65% wheat flour, 20% maize flour, and 15% BSG flour.^121^

Fermentation emerges as a valuable method for enhancing ingredient functionality and bread quality. It extends microbial shelf life, refines dough characteristics, minimizes staling, and boosts specific volume, contributing to overall product improvement.^133^ Another approach to enhance BSG addition in bread is BSG fermentation by commercial enzymes or microbial fermentation. The evaluation of commercial enzymes, amylase, xylanase, and endoglucanase, demonstrated their effectiveness in enhancing white wheat bread’s structure and loaf volume (WB). These enzymes improved texture, increased loaf volume, and extended shelf life, with the appropriate enzymatic treatment and BSG incorporation.^93^ The enzymatic hydrolysis (commercial cellulase) of BSG (12%) in an extruder was evaluated as a bioconversion process to improve flour for bread making. After 24h, bread produced with BSG + 1% cellulase showed less hardness (6.29 N) than BSG not enzymatic treated, no difference was observed in bread elasticity (0.91). The sensorial panel demonstrated an equal preference for the investigated formulations, indicating that despite the efficient modification of the BSG and bread characteristics, the preference for bread produced from treated and untreated flours did not differ.^134^

Sourdough fermentation is widely used in bread making to enhance the palatability of cereal brans and wholemeal flour. A sourdough starter improves bread volume, texture, and shelf life, influencing bioactive compound levels, including phytate, folates, tocopherols, and phenolic compounds. The use of *L. brevis*, *L. plantarum*, and *S. cerevisiae* in BSG bread demonstrated significant benefits, such as a reduction in phytic acid levels by more than 30% compared to the unfermented counterpart, and an increase in antioxidant activity by up to 36%. Additionally, cellulolytic and hemicellulolytic hydrolases, including xylanases, act on nonstarch polysaccharides (NSP), improving dough handling and enhancing baked product volume. Xylanase and dough conditioners further optimize bread’s specific volume, texture, and sensory attributes while influencing staling kinetics. Sourdough addition did not affect loaf specific volume (2.2 minmL/g). However, the specific volume was significantly improved with the addition of xylanase and dough conditioner (2.7 mL/g). Sourdough fermentation also impacts the final aroma of BSG bread by releasing amino acids essential for forming volatile compounds.^92^

Biocatalysts created by immobilizing baker’s yeast, kefir, or *Lactobacillus casei* cells on BSG were found to be effective in bread making, whether using the straight dough or sourdough method, as a substitute for conventional pressed baker’s yeast. When 6, 8, and 10% w/w (flour basis) of baker’s yeast or kefir immobilized on BSG was mixed with wheat flour, the bread rose well, improved overall quality, enhanced flavor, and remained fresh for a longer period. Sourdough bread exhibited superior results, including higher moisture retention during baking, reduced water evaporation, slower staling rates, and extended freshness (4–5 days). Additionally, the higher acidity of sourdough bread and potential bacteriocin formation contributed to a shelf life twice as long as that of traditional baker’s yeast bread. Consumer evaluations indicated a stronger preference for sourdough bread regarding aroma, taste, and overall quality. Furthermore, BSG served as a substrate for immobilizing *L. casei*, suggesting its potential as a prebiotic that promotes lactic acid bacteria growth.^135^

Bread made with BSG flour fermented using *L. plantarum* was well received for its structural and sensory qualities. Fermentation contributed to a softer texture and enhanced springiness. Fermented BSG substituted at the lower level resulted in softer bread than the wheat bread control (4.71 N), whereas up to 15% substitution levels resulted in breads that were softer (8.03 N) than 5–10% BSG (5.33–6.27 N) or compared to 15% the wholemeal control bread (11.62 N). To produce acceptable bread, levels of 15% BSG or BSG SD supplementation should not be exceeded. The 10% substituted bread was well accepted, thanks to BSG’s nutritional benefits, especially its protein, dietary fiber, and mineral content. This makes BSG an effective fortifying ingredient, improving texture and dough handling, particularly after fermentation with *L. plantarum* FST 1.7.^136^

Various levels of BSG incorporated into white and whole wheat flour influenced dough rheology and were assessed for their impact on bread quality and antioxidant properties through sourdough fermentation. BSG was dried at 150 °C for four hours and finely milled to 200 and 125 μm. Wheat flour blends containing 0%, 10%, 15%, and 20% BSG underwent fermentation using commercial strains of *L. plantarum* and *L. brevis* for sourdough preparation. Increasing BSG levels resulted in a notable decrease in specific loaf volume and increased crumb hardness. The crumb hardness increased significantly when the BSG (1417–2070 g, for 10 and 20% BSG) was incorporated into the wheat flour (846.4 g), In case of the samples with sourdough (1203.3–1468.4 g, for 10–20% BSG), the crumb hardness was lower with respect to the corresponding samples without sourdough. However, sourdough fermentation improved overall bread characteristics while enhancing total phenolic content and antioxidant activity in BSG-enriched bread.^137^

Wheat bread was enriched with spray-dried BSG and fermented BSG (FBSG). Including FBSG notably reduced dough stiffness and had a milder impact on gluten network formation than unfermented BSG. These dough modifications enhanced the bread’s techno-functional properties, increasing specific volume while reducing crumb hardness. The softest crumb was determined in the baker’s flour bread—BF (2.99 ± 0.36 N), while the wholemeal flour bread showed a significantly harder crumb (30.13 ± 6.15 N). The replacement of BF by BSG and fermented FBSG at a source of fiber level increased crumb hardness to 10.91 ± 1.32 N and 7.91 ± 1.31 N, respectively. The increase in inclusion level of BSG and FBSG amplified the elevation in crumb hardness, resulting in the highest values (BSG HF: 79.22 ± 5.88 N; FBSG HF: 47.24 ± 3.97 N). Comparing BSG and FBSG with each other, FBSG caused a softer crumb. Beyond improving bread quality, fermenting BSG with *L. plantarum* f10 and/or *L. rhamnosus* GG (LGG) yielded an ingredient that extended microbial shelf life and slowed bread staling. Among the formulations tested, BF bread exhibited the fastest staling rate (2.10 ± 0.49). In contrast, WMF bread showed a reduced staling rate (1.0 ± 0.12). Substituting BF with BSG and FBSG further decreased the rate to 1.34 ± 0.35 and 1.73 ± 0.13, respectively. Higher levels of BSG and FBSG addition led to an even greater reduction (BSG HF: 0.70 ± 0.14; FBSG HF: 1.08 ± 0.06). When comparing BSG and FBSG formulations, BSG breads consistently showed slightly lower staling rates; however, these differences were not statistically significant.^133^

Milling and bioprocessing techniques, particularly fermentation, offer promising pathways for integrating more BSG into food applications, enhancing their nutritional and functional benefits. A deeper understanding of these processes can assist researchers, businesses, and bread manufacturers in developing and refining products that align with consumer preferences while promoting the utilization of industrial byproducts in bread production.

Weiss beer is another product of microbreweries, and the byproduct wheat BSG can also be used to produce bread. Steamed bread was made with WBSG and wheat flour blended at 2.5% to 20%. The dough was prepared with yeast fermentation, and the bread was steamed with boiling water for 25 min. WBSG significantly enhanced pasting viscosities, pasting temperature, and the proportion of immobilized water in doughs, demonstrating its potential to improve dough structure and functionality. Sensory evaluation of appearance and taste properties showed that pleasant malt flavor and chocolate-colored appearance in steamed bread were acquired when the WBSG substitution level was less than 5%.^138^

Biscuits, traditionally made from wheat flour, tend to be high in calories but low in protein and fiber. BSG, being rich in both nutrients, is an excellent fortifier for enhancing the nutritional profile of biscuits. After drying to a moisture level of 10% and milling, BSG flour is incorporated into biscuit recipes. Adding 10% BSG contributed to a fragrant wheat aroma and a chocolate-like appearance, improving sensory and visual attributes.^122^ Sensorial acceptance of BSG-fortified biscuits was tested by mixing wheat flour, oat, and BSG flour, indicating that ingredient manipulations affected the sensory characteristics and consumers’ preferences of the BSG-fortified biscuits. Different clusters of young consumers had different preferences and different textural perceptions of these biscuits.^123^

Cookies supplemented with BSG of medium (212–425 μm) and coarse (425–850 μm) particle sizes demonstrated strong potential as a dietary fiber source. BSG preparations of coarse, medium and fine particle size were added into the cookie formulations at the level soft 5, 10, 15, 20 and 25%. BSG with larger particle sizes yielded better cookie properties than fine particulate BSG. Sensory evaluations revealed that lower levels of medium and coarse BSG substitutions produced cookies rated at least as favorably as the control samples. Panelists accepted cookies with up to 15% BSG (medium and corse) addition, while the acceptable level of supplementation for the fine (<212 μm) particle size BSG was determined to be as low as 5%.^11^

Partially replacing wheat flour with BSGs in shortbread formulations offers an effective strategy for upcycling and enhancing BSG’s value in functional food production. Substituting 30% wheat flour with BSGs barley malt flour and BSG enriched with 30% oat flakes significantly boosts fiber (especially arabinoxylan) and protein content. This adjustment leads to a 10-fold increase in total dietary fiber (TDF), qualifying the experimental shortbreads as “high in fiber.” The panelists highlighted significant differences with the shortbread based on 100% wheat flour, such as a darker color, more pungent odor, grainy and toasted texture, and weaker odor and taste of sugar, butter, and egg. The shortbread formulated by partially substituting wheat flour with BSGs could meet consumer compliance because of its high nutritional value, sustainability, and acceptable sensory characteristics.^139^

### 6.2 Application in Pasta Making

Dry pasta is a global staple food, thanks to its affordability, versatility, extended shelf life, and balanced sensory and nutritional qualities. Its adaptability allows for endless culinary possibilities, making it a fundamental ingredient in various cuisines worldwide. Sheets for lasagna with BSG flour and fiber-enriched fresh egg were prepared. BSG was dried in a vacuum oven at 60 °C for 48 h and milled to a size <500 μm. The egg was added to the pasta recipe as liquid, as was pasteurized whole egg and egg white powder (EWP). Thickness was measured using a caliper. Mechanical properties were assessed through tensile tests performed with an Instron Universal Testing Machine 3365 (Instron Division of ITW Test and Measurement Italia S.r.l., Trezzano sul Naviglio, Italy), equipped with a 100 N load cell. Analyses were conducted on dumbbell-shaped samples at room temperature, with a constant crosshead speed of 20 mm/min. The increase in pasta weight due to cooking was determined by weighing pasta sheets before and after boiling. Cooking loss was evaluated by measuring the dry matter content of the residual cooking water, after restoring it to the initial volume with natural spring water.

The key quality attributes of pasta are closely linked to its cooking performance and the mechanical properties of both raw and cooked samples. Each specimen was boiled for 3 min under standardized conditions, maintaining a fixed pasta-to-water ratio of 1:10 (w/v). Pasta sheets produced with the addition of BSG exhibited a significantly lower average thickness compared to the standard formulation, both before cooking (1.13 ± 0.03 vs 1.29 ± 0.02 mm) and after cooking (1.33 ± 0.09 vs 1.52 ± 0.07 mm). This reduction can be attributed to the decreased elasticity of the dough caused by BSG incorporation. Although the optimized formulation contained only a modest amount of BSG (6.2%), this finding is particularly relevant for breweries, as BSG valorization enhances the sustainability of the brewing process. Dry pasta is widely recognized as a staple food due to its affordability, versatility, long shelf life, and balanced sensory and nutritional attributes. Its ability to pair with a wide range of flavors makes it an indispensable ingredient in diverse culinary traditions worldwide.^124^

Incorporating egg white powder (EWP) was assessed for enhancing pasta structure, while BSG was utilized to enrich durum wheat semolina, increasing its nutritional potential. BSG dried at 60 °C for 72 h and was micronized to ≤700 μm. Adding micronized BSG to semolina significantly boosted total dietary fiber, β-glucan, resistant starch, and total antioxidant capacity compared to the control. Sensory and instrumental evaluations indicated that spaghetti containing 5% BSG displayed textural properties similar to traditional durum semolina pasta, particularly regarding overall quality and firmness. However, 10% BSG emerged as the optimal balance, offering the best combination of technological performance, nutritional benefits, and sensory attributes.^140^

BSG, derived from lager beer production using 70% barley malt and 30% maize (*Zea mays*), underwent xylanase treatment followed by fermentation with *L. plantarum* PU1. Bioprocessed BSG (15%) was incorporated into fortified semolina pasta, qualifying it for “high fiber” and “source of protein” labeling under European Community Regulation No. 1924/2006. Compared to native BSG, bioprocessed BSG demonstrated superior protein digestibility and improved nutritional indices, including essential amino acid index, biological value, protein efficiency ratio, and nutritional index, while lowering the predicted glycemic index. Additionally, bioprocessing enhanced the technological properties of fortified pasta, resulting in a distinctive sensory profile markedly enriched by the pretreatment, reinforcing its potential as a health-promoting food ingredient.^141^

Two BSG-derived ingredients were incorporated into semolina-based pasta formulations. Rheological assessments determined the optimal ingredient proportions, resulting in pasta that meets the “High Protein” and “High Fiber” claims with 15% protein-rich BSG, and the “High Fiber” and “Source of Proteins” claims with 10% fiber-rich BSG. These formulations were evaluated against 100% semolina and 100% whole grain semolina pasta for composition, color, texture, and cooking quality, demonstrating excellent performance. The newly developed pasta achieves a balance of technological efficiency, nutritional and sensory quality, and sustainability.^142^

### 6.3 Application for Cereal Snacks

As the demand for healthier and calorie-reduced snacks grows, the food industry faces the challenge of maintaining taste while enhancing nutritional value. BSG has proven to be a valuable ingredient for baked and extruded snacks, offering antioxidant, fiber, mineral, and essential fatty acid enrichment. Addition of 5%, 10%, and 15% BSG in corn grits snacks significantly affected protein content, fiber composition, and texture. Extrusion (at screw compression ratio: 4:1; round die head 4 mm nozzle diameter; temperature profile: 135 °C in dosing zone, 170 °C in compression zone and 170 °C in ejection zone; screw speed: 100 rpm; dosing speed: 20 rpm) reduced insoluble fiber while increasing soluble fiber, though BSG addition led to a lower expansion ratio and fracturability, while bulk density and hardness increased. The extrusion process had a significant effect on the increase of water absorption index (WAI) and water solubility index (WSI). Corn grits +5% BSG showed an increase of 2.58  to 7.15g/g for WAI and 3.13 to 17.63% for WSI, after extrusion. The introduction of 1% pectin effectively mitigated expansion issues in BSG-containing extrudates. Among tested formulations, snacks with 5% BSG received the highest scores for external appearance, texture, consistency, odor, and flavor, highlighting their potential for improving snack quality while boosting nutritional benefits.^125^

The nutritional benefits of BSG in cereal bakery products have been explored, particularly in breadsticks. Incorporating 25% and 35% BSG significantly elevated protein levels, while including 15% BSG more than doubled the dietary fiber content, demonstrating its potential as a valuable fortifier in snack formulations.^126^ For baked snacks (crispy slices), BSG flour (moisture 5.6%), a percentage of 10% BSG received the highest mean scores in all quality attributes (texture, flavor, aroma, appearance), and overall acceptability. A decrease in acceptability was observed with increased BSG addition levels; in particular, 25% of BSG-containing samples were the least acceptable.^101^

A consumer-led study explored perceptions of cereal-based snack concepts featuring BSG as a sustainable and functional ingredient. Findings highlighted crispy crackers as the most promising option for further development, followed by crispy sticks with dip, fruity biscuits, and twisted breadsticks. While consumers demonstrated environmental awareness concerning packaging choices, the broader ecological advantages of incorporating BSG into foods were not necessarily seen as a driving factor in purchasing decisions, which underscores the importance of manufacturers in designing sustainable snacks while effectively communicating their environmental benefits to consumers.^99^

### 6.4 Application for Muffins

The impact of two drying techniques,impingement and hot-air drying, on the chemical composition, physicochemical properties, and bioactive compounds of BSGs in muffins was examined. Muffins fortified with 15% and 20% impingement-dried BSG improved fat, protein, dietary fiber, and bioactive compound levels. However, the batter fortified with 15% BSG demonstrated lower viscosity and retained a brighter muffin color than the 20% variant, potentially enhancing consumer appeal. Consequently, the 15% BSG-fortified sample was selected for sensory evaluation, aiming to introduce consumers to value-added products and assess their interest in such innovations.^127^ In another study, a sensory analysis of muffins fortified with BSG flour revealed strong consumer acceptance for formulations with higher BSG content. Muffins containing 20% BSG received the highest overall ratings, while those with 30% BSG, despite their elevated protein, fiber, and antioxidant levels, still achieved acceptable scores across all evaluated attributes. Increasing BSG concentration can enhance nutritional benefits while maintaining favorable sensory qualities.^117^

Muffins incorporating BSG, as well as BSG treated with commercial enzymes demonstrated promising results. Biocellulase A, Bioglucanase FS2000 and Bioglucanase HAB, were added to BSG (75 μL g^–1^ dw) and the reaction was maintained for 1 h at 50 °C. The resulting suspension was then adjusted to pH 9.30 with 30% NaOH (w/v) and it was allowed to incubate for 2 h at 50 °C with Alcalase 2.4 L (2%, v/w, BSG protein), after which Bioprotease FV was added (1% v/w, BSG protein). Following a further 2 h incubation at 50 °C, and then the suspension was heat-inactivated. Substituting up to 10% BSG had no negative impact on sensory attributes related to appearance and texture while significantly enhancing fiber content compared to the control. In contrast, the substitution of unhydrolyzed BSG showed no notable effect at levels up to 5%. This suggests enzymatic treatment may optimize BSG’s contribution to muffins’ texture and nutritional value.^143^

In addition to the percentage of BSG, the formulation of ingredients can also influence muffin acceptability. In addition to flour, eggs, milk, salt, sugar, and oil, ordinary ingredients in muffins, the well-accepted 20% BSG muffin presented by Combest and Warren (2022) includes muffin recipe ingredients such as vanilla extract and ground cinnamon. Although the studies did not mention sensory analysis of the products presented below, BSG has also been applied to producing meat-based snacks, yogurt, and fermented drinks.

### 6.5 Application for Meat Products

BSG can be added as a novel ingredient in meat analogs to improve their texture by reducing hardness and enhancing nutritional value by increasing fiber content. BSG was added to soy protein-based high-moisture meat analogues (HMMAs). Incorporating 15% BSG into high-moisture meat analogs (HMMAs) improved texturization and reduced hardness. However, higher levels hindered the development of a fibrous structure replicating animal-based products’ mouthfeel. Including BSG in soy protein-based meat alternatives presents a promising approach for expanding the diversity of plant-based meat products while advancing sustainability and nutritional benefits.^10^

Adding BSG and mechanically deboned poultry meat (MDPM) into corn-based extruded snack products aims to create nutritionally valuable products desirable to children and adults, which could contribute to promoting and facilitating. Adding protein- and fiber-rich ingredients creates a nutritionally fortified snack product with lower-quality physical and textural characteristics and altered color. The optimized snack formulation contained 4% MDPM and 14.8% BSG, and it was produced at 900 rpm screw speed.^100^

Hybrid sausages incorporating broccoli, BSG, and *T. molitor* insect flour (IF) offer an innovative approach to diversifying protein sources while reducing reliance on animal-based ingredients. The formulation containing 22% broccoli, 3% BSG, and 10% IF in turkey-based sausage demonstrated juiciness and odor comparable to conventional sausage products, with notable improvements in appearance. This strategy highlights the potential BSG in creating sustainable and nutritious protein source.^128^ Pro-environmental behaviors related to protein consumption emphasize “using fewer natural resources” and “adopting practices that reduce environmental impact.” Practical examples include reducing meat portion sizes, enhancing their nutrient density, and substituting meat with plant-based protein sources.^144^

The impact of substituting pork back fat with BSG pre-emulsion in chicken sausages was assessed. The pre-emulsion, prepared using 15% BSG dietary fiber extracts, was incorporated at 20%, 25%, and 30% replacement levels. Results indicated that BSG pre-emulsion enhanced the’ hardness, gumminess, and chewiness. Notably, replacing 20–25% of pork back fat with BSG pre-emulsion proved effective in improving the texture and overall quality of the sausages.^129^

### 6.6 Application for Beverage Production

Soy-based yogurt alternatives enriched with BSG and protein hydrolysates were developed through fermentation involving *Streptococcus thermophilus*, *Lactobacillus delbrueckii* ssp. bulgaricus, *Lactobacillus acidophilus*, and *Bifidobacterium lactis*. Microbial culture (0.5 g/kg) was added into the mixture BSG and Water-soluble soybean extracts (WSE), ratio 1:9, BSG-WSE (g/g) and the temperature was kept at 43 °C to reach pH range at 4.3–4.7. The inclusion of BSG derivatives influenced microstructural formation during fermentation, helping to maintain flow behavior and consistency throughout storage. This suggests that BSG could be valuable in enhancing texture and stability in plant-based yogurt formulations. During the storage (14 days), the acidity remained stable. The fluctuation of pH and lactic acid during the storage might be due to the fluctuation in the survival of lactic acid bacteria, although the amount of lactic acid bacteria was not included in this study.^145^

Unlike previous reported studies that primarily utilized solid BSG waste, a research explored the potential of a fermented beverage derived from its liquid fraction. A plant-based yogurt alternative was formulated using a commercial soy drink and BSG liquid fermented with plant-adapted lactic acid bacteria. The BSG liquid (LBSG) was filtered, and a 100 μm fraction was blended with commercial unsweetened soy drink (SoD) in a 20:80-LBSG-soy drink ratio, and the pH was adjusted to 7.0. The performance of commercial lactic acid bacteria in plant-based fermentations has often been reported as suboptimal, prompting an initial screening. A total of 171 strains were tested on agar, of which 96 heterofermentative strains produced an intense yellow halo, indicative of acid formation, when cultivated in LBSG:SoD medium at a 25:75 (v/v) ratio. Eight strains with the highest lactic acid production, belonging to three species, such as *Leuconostoc mesenteroides*, *L. plantarum*, and *L. lactis*, were selected. *Leuconostoc* species generated considerable amounts of ethanol, which is undesirable in yogurt production. The facultative heterofermentative species *L. lactis* and *Lb. plantarum* also exhibited mixed acid metabolism, though at lower levels compared to *Leuconostoc*. Fermentation was ultimately carried out using a combination of *L. lactis* and *Lb. plantarum* (2:1). The resulting product displayed a protein content of 3.59 ± 0.01 g/100 mL and a pH of 4.59 ± 0.02, values slightly higher than those observed in commercial soy-based yogurt. The final product’s content and properties were compared to a commercial plant-based yogurt-like product and a dairy yogurt. Findings revealed that fermenting a soy drink with 20% BSG liquid fraction resulted in a texture and sensory profile closely resembling traditional dairy yogurt, highlighting its potential as a sustainable and functional alternative.^146^

Cow milk with 5% to 20% of BSG previous pasteurized (90 °C for 15 min) were fermented with 0.05% (w/w) microbial culture (*S. thermophilus*, *L. delbrueckii* ssp bulgaricus, *L. acidophilus*, and *B. lactis*) at 38–43 °C. pH range 4.3 and 4.8 was achieved after 3–4 h of fermentation. Then the fermentation was ended by homogenizing using a laboratory scale mixer at a higher speed for 10 s (380 rounds/min; 4 cm gap) and cooling down to 15 °C. The yogurt was stored at 4 °C and the analyses were conducted at 1, 7, and 14 days of storage. The incorporation of BSG in yogurt production significantly affected (*p* < 0.05) the quantity of lactic acid bacteria (LAB) during storage. In the control group (0% BSG), the number of *S. thermophilus* *and* *L. bulgaricus* increased throughout the observed period, while the addition of BSG caused fluctuations in these microorganisms. Despite this, at the end of the experiment there was no significant difference (*p* > 0.05) between the groups. These results indicate that the growth of LAB in yogurt is unstable in the presence of BSG, but without a negative impact on its viability during storage. BSG 5–10% optimizes key quality parameters, including acidity, rheological behavior, and lactic acid bacteria (LAB) growth in yogurt milk. While a 15%–20% substitution-maintained acidity and LAB levels and minimized syneresis, it negatively impacted yogurt’s flow performance. This suggests moderate BSG incorporation can enhance yogurt’s functional and nutritional attributes while preserving its desirable texture.^131^

BSG flour (BSGF) and protein extracts (BSGPs) were incorporated into water-soluble coconut extract (WSCE) at a 1:9 (w:w) ratio, using the same microbial mix and fermentation conditions previously described by Naibaho et al.^131^ The quantity of lactic acid bacteria (LAB) was not determined in this study. The time required to reach the target pH range varied depending on the type of BSG derivative. The addition of BSGF resulted in a pH of 4.5–4.7 after 3 h of incubation, similar to the control. In contrast, all BSGPs required a longer incubation time (4–4.5 h) to achieve the same pH range. These differences were influenced by the initial pH: while BSGF did not alter the starting pH compared to the control, BSGPs increased the initial pH of the mixture. About rheological parameters, the fermentation of BSG flour (BSGF) and protein extracts (BSGPs) enhanced mechanical properties, viscosity, and flow behavior, ensuring stable texture and gel formation. Even after 14 days of storage, key attributes such as flow behavior, syneresis, and mechanical integrity remained well-preserved.^130^

Submerged fermentation of BSG using *B. subtilis* WX-17 (10^6^ CFU/g) was carried out for 72 h at 37 °C, 200 rpm, without supplementary components, yielded a novel nutritional beverage. The changes in *B. subtilis* WX-17 cell count (CFU/mL) were recorded over 6 weeks at 4 °C. The count decreased slightly across 6 weeks with 10.48 log CFU/mL in week 0, to 9.86 log CFU/mL after 6 weeks. The beverage exhibited elevated amino acid levels, total phenolic content, antioxidant activity, and bioactive compounds. This combination of enhanced nutritional properties and sustained bacterial viability underscores its potential as a health-promoting beverage.^132^

## 7 Conclusion

The growing microbrewery sector highlights the importance of addressing waste management challenges in beer production. This study delved into solid waste generation, characterization, and environmental impacts such as BSG, BSY, and hot trub. Though often considered waste, these byproducts hold significant potential for innovative applications, particularly in the food industry. The integration of conservation technologies has proven essential for extending the shelf life and ensuring the safety of these materials, enabling their use in diverse food applications. Particularly for BSG, numerous examples include its incorporation into baked goods, pasta, cereal snacks, meat products, and beverages. These applications add value to brewery residues and align with sustainability principles by promoting resource efficiency and waste reduction.

A clear operational plan is required to apply the insights from this analysis to small breweries. Stabilization choices should be aligned with each brewery’s budget and space constraints, ranging from low-cost options, such as immediate chilled storage of wet BSG, which often requires 2–3 m^2^ of floor space per 1000 L of beer and may demand 15–25 kWh/day for drying, to intermediate alternatives like cabinet dryers or simple fermentation units that extend residue shelf life. Byproduct valorization pathways can also be implemented, including the production of dried BSG flour (yielding roughly 180–220 g per kg of wet BSG), BSY protein extracts, or biogas generation via anaerobic digestion, which can convert organic residues into usable energy while reducing disposal costs. Finally, collaborative models, such as shared dryers, regional processing hubs, or community-scale anaerobic digesters, can offer cost-effective solutions for microbreweries operating with limited space and resources. Incorporating these elements provides a structured and realistic path for microbreweries to begin implementing circular economy strategies and move toward more resilient, resource-efficient, and economically viable waste valorization systems.
